# LASSI: A lattice model for simulating phase transitions of multivalent proteins

**DOI:** 10.1371/journal.pcbi.1007028

**Published:** 2019-10-21

**Authors:** Jeong-Mo Choi, Furqan Dar, Rohit V. Pappu

**Affiliations:** 1 Department of Biomedical Engineering, Washington University in St. Louis, St. Louis, MO, United States of America; 2 Center for Science & Engineering of Living Systems (CSELS), Washington University in St. Louis, St. Louis, MO, United States of America; 3 Department of Physics, Washington University in St. Louis, St. Louis, MO, United States of America; Koç University, TURKEY

## Abstract

Many biomolecular condensates form via spontaneous phase transitions that are driven by multivalent proteins. These molecules are biological instantiations of *associative polymers* that conform to a so-called *stickers*-and-*spacers* architecture. The stickers are protein-protein or protein-RNA interaction motifs and / or domains that can form reversible, non-covalent crosslinks with one another. Spacers are interspersed between stickers and their preferential interactions with solvent molecules determine the cooperativity of phase transitions. Here, we report the development of an open source computational engine known as LASSI (**LA**ttice simulation engine for **S**ticker and **S**pacer **I**nteractions) that enables the calculation of full phase diagrams for multicomponent systems comprising of coarse-grained representations of multivalent proteins. LASSI is designed to enable computationally efficient phenomenological modeling of spontaneous phase transitions of multicomponent mixtures comprising of multivalent proteins and RNA molecules. We demonstrate the application of LASSI using simulations of linear and branched multivalent proteins. We show that dense phases are best described as droplet-spanning networks that are characterized by reversible physical crosslinks among multivalent proteins. We connect recent observations regarding correlations between apparent stoichiometry and dwell times of condensates to being proxies for the internal structural organization, specifically the convolution of internal density and extent of networking, within condensates. Finally, we demonstrate that the concept of saturation concentration thresholds does not apply to multicomponent systems where obligate heterotypic interactions drive phase transitions. This emerges from the ellipsoidal structures of phase diagrams for multicomponent systems and it has direct implications for the regulation of biomolecular condensates *in vivo*.

## Author summary

Spatial and temporal organization of molecular matter is a defining hallmark of cellular ultrastructure and recent attention has focused on membraneless organelles, which are also referred to as biomolecular condensates. Of interest are condensates that form via phase transitions that combine phase separation and networking of multivalent protein and nucleic acid molecules. Building on recently recognized analogies between associative polymers and multivalent proteins, we have developed and deployed LASSI, an open source computational engine that enables the calculation of architecture-specific phase diagrams for multivalent proteins. LASSI relies on *a priori* identification of stickers and spacers within a multivalent protein and mapping the stickers onto a 3-dimensional lattice. A Monte Carlo engine that incorporates a suite of novel and established move sets enables simulations that track density inhomogeneities and changes to the extent of networking among stickers as a function of protein concentration and interaction strengths. Calculation of distribution functions and other order parameters allow us to compute full phase diagrams for multivalent proteins modeled using a *stickers*-and-*spacers* representation on simple cubic lattices. These calculations allow us to rationalize experimental observations and open the door to the design of protein architectures with bespoke phase behavior. LASSI can be deployed to study the phase behavior of multicomponent systems, which allows us to make direct contact with the physical principles underlying cellular biomolecular condensates.

## Introduction

Biomolecular condensates organize cellular matter into non-stoichiometric assemblies of proteins and nucleic acids [1]. Prominent condensates include nuclear bodies [2] such as nucleoli, nuclear speckles [3, 4], and germline granules [1, 5, 6]. Condensates also form in the cytoplasm. These include stress granules [7], membrane-anchored signaling clusters [8, 9], and bodies in post-synaptic zones [10]. All of these condensates share key features: (i) they range in size from a few hundred nanometers to tens of microns [1, 2, 11]; (ii) they are multicomponent entities comprising of hundreds of distinct types of proteins and nucleic acids; (iii) and of the hundreds of different types of molecules that make up condensates, a small number are essential for the formation of condensates [1, 12]. The simplest feature that distinguishes proteins that are drivers of biomolecular condensates is the valence of interaction domains / motifs that can participate in non-covalent crosslinks [1, 12–14].

Biomolecular condensates can form and dissolve in an all-or-none manner [2, 11, 15]. The reversible formation and dissolution of condensates can be controlled by the concentrations of multivalent proteins that drive the formation of condensates; in simple two-components systems comprising of macromolecules and solvent, condensates form when macromolecular concentrations cross macromolecule-specific threshold values known as *saturation concentrations* [15]. The transitions that characterize condensate formation bear the hallmarks of a sharp transition in macromolecular density, leading to the formation of a dense phase that is in equilibrium with a dilute phase. This type of transition, known as *phase separation*, sets up two or more coexisting phases to equalize the dense and dilute phase chemical potentials of the macromolecules across phase boundaries [15]. Phase separation is reversible and this reversibility can be achieved by (i) changes to concentrations of the driver macromolecules [9, 16], (ii) changes to solution conditions that alter the effective interaction strengths among driver molecules [17–20], (iii) altering saturation concentrations through ligand binding–a phenomenon known as polyphasic linkage [21, 22], or (iv) via biological regulation such as post-translational modifications of proteins [8, 12, 23].

Recent studies have focused on uncovering the defining features of proteins [13, 15, 17–19, 24–40] and RNA molecules [41–43] that drive phase transitions. Protein and RNA molecules that drive phase transitions are biological instantiations of *associative polymers* [44] characterized by a *stickers*-and-*spacers* architecture [45]. Stickers contribute to a hierarchy of specific pairwise and higher-order interactions that are either isotropic or anisotropic whereas spacers control the concentration-dependent inhomogeneities in the densities of stickers around one another. Stickers can be *hot spots* or *sectors* [46] on the surfaces of folded proteins [15, 29] or short linear motifs within intrinsically disordered regions (IDRs) [15, 24, 47]. Spacers are typically IDRs that contribute through their sequence-specific effective solvation volumes to the interplay between *density transitions* (phase separation) and *networking transitions* that are better known as percolation [28, 29]. Spacers can also be folded domains that are akin to uniformly reactive colloidal particles, although this has not yet been explored. Proteins can be mapped onto the stickers-and-spacers architecture as linear multivalent proteins, branched multivalent proteins, or some combination of the two [13, 15].

Simple two-component systems comprise of the solvent (which includes all components of the aqueous milieu) and a multivalent protein / RNA molecule. For fixed solution conditions, one can generate phase diagrams [25] as a function of protein concentration, the valence of stickers, the affinities of stickers, the sequence-specific effective solvation volumes of spacers, and the lengths / stiffness of spacers. The phase diagram can be investigated by keeping the valence of stickers, the lengths of spacers, and effective solvation volumes of spacers fixed while varying the concentration of stickers and the affinities between stickers [29]. Changes to protein concentration will enable density fluctuations and above the saturation concentration, designated as *c*_sat_, the density inhomogeneities lead to separation of the system into coexisting phases. The concentration of multivalent proteins in the dilute and dense phases will be denoted as *c*_sat_ and *c*_dense_, respectively. For a given bulk concentration *c*_bulk_ that lies between *c*_sat_ and *c*_dense_, the fraction of molecules within each of the coexisting phases is governed by the lever rule [48].

Stickers also form reversible physical crosslinks and these crosslinks generate networks of inter-connected proteins. The number of proteins within the largest network of the system grows continuously as the protein concentration increases. Above a concentration threshold known as the percolation threshold and designated as *c*_perc_, the single largest network spans the entire system and this phenomenon is called *percolation* [49–51]. If the percolated networks have the rheological properties of viscoelastic fluids, the fluids are referred to as *network fluids* [15, 52].

**Fig 1:**
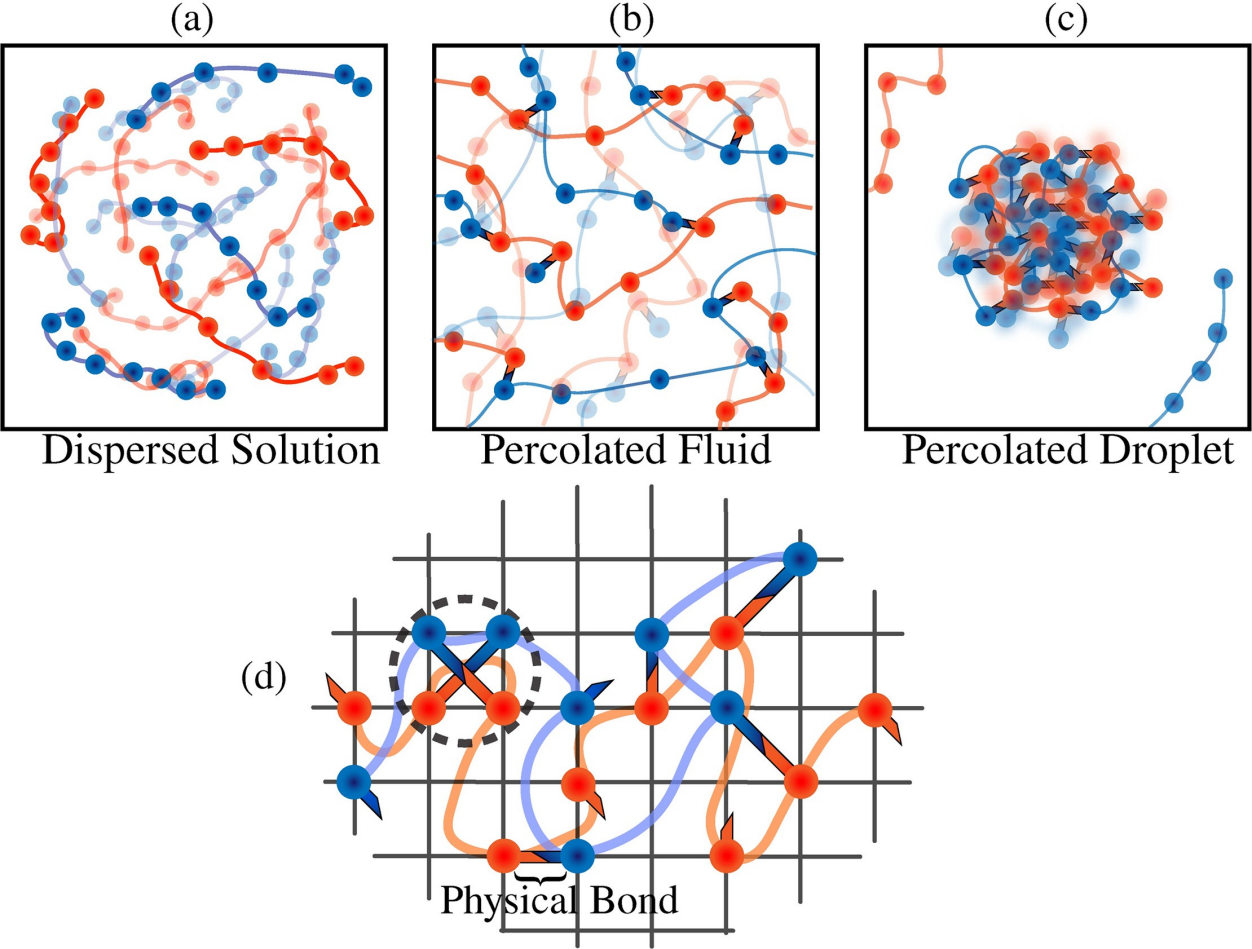
Characteristic phases in the stickers and spacers formalism. (a) Dispersed solution phase where the polymers are uniformly mixed in solution. (b) Percolated fluid wherein the polymer chains form a percolated, system-spanning network through physical crosslinks among stickers results. (c) Droplet wherein network formation also causes the polymers to form condensed phases. (d) Two-dimensional representation of the LASSI architecture. The beads with arms denote stickers where arms denote that the monomers are capable of orientational interactions, and the curved lines connecting the monomers represent phantom tethers, which are allowed to freely overlap (implicit spacer model). Different colors denote different sticker and spacer species respectively. Note that the physical bonds are allowed to overlap (dashed circle). For the rest of this work, physical bonds will not be labeled and will only be depicted as overlapping orientational arms.

Phase separation and percolation can be coupled to one another. The coupling will depend on the values of *c*_sat_, *c*_dense_, and *c*_perc_ relative to *c*_bulk_. If *c*_bulk_ is smaller than all of *c*_sat_, *c*_dense_, and *c*_perc_, the system is in a single dilute phase with no large molecular networks (**Fig 1A**). If *c*_bulk_ > *c*_perc_ and *c*_perc_ < *c*_sat_, then a system-spanning percolated network forms without phase separation (**Fig 1B**). However, the system undergoes phase separation and a dense phase forms as a *percolated droplet* if *c*_bulk_ > (*c*_sat_, *c*_perc_) and *c*_sat_ < *c*_perc_ < *c*_dense_ (**Fig 1C**). Recent studies, using three-dimensional lattice models designed to mimic the poly-SH3 and poly-PRM systems of Li *et al*. [16], show that sequence-specific effective solvation volumes of linkers / spacers between folded domains directly determine whether phase separation and percolation are coupled or if percolation occurs without phase separation for linear multivalent proteins [28, 29]. The coupling between phase separation and percolation is controlled by the extent to which spacers / linkers preferentially interact with the surrounding solvent.

Theory [17, 24, 25, 27, 34, 53–59] and computations [28, 29, 43, 60–68] have important roles to play in modeling and describing the phase behavior of multivalent protein and RNA molecules. Theories provide analytical routes to explain experimental observations and to make testable predictions. On the other hand, simulations work around many of the simplifying assumptions that are needed to make theories analytically tractable. In doing so, they provide numerical routes to enable comparative assessments across different systems; they help in making testable predictions about phenomenology through *what if* calculations targeted toward specific systems; and they pave the way for designing systems with bespoke phase behavior.

Phase transitions are collective phenomena that involve highly cooperative transitions of large numbers of multivalent polymers. The collective interactions that drive phase transitions are captured in terms of a small number of order parameters that are similar across disparate systems and represent a generic coarse-graining of the underlying system that defines parameters such as the correlation length and the sizes of cooperative units. Accordingly, practical considerations of computational tractability and rigorous considerations of identifying the relevant collective coordinates mandate the use of coarse-grained models for simulations of phase transitions driven by multivalent protein and RNA molecules. We focus here on multivalent proteins, although the methods we describe are readily adaptable to RNA molecules as well.

**Fig 2:**
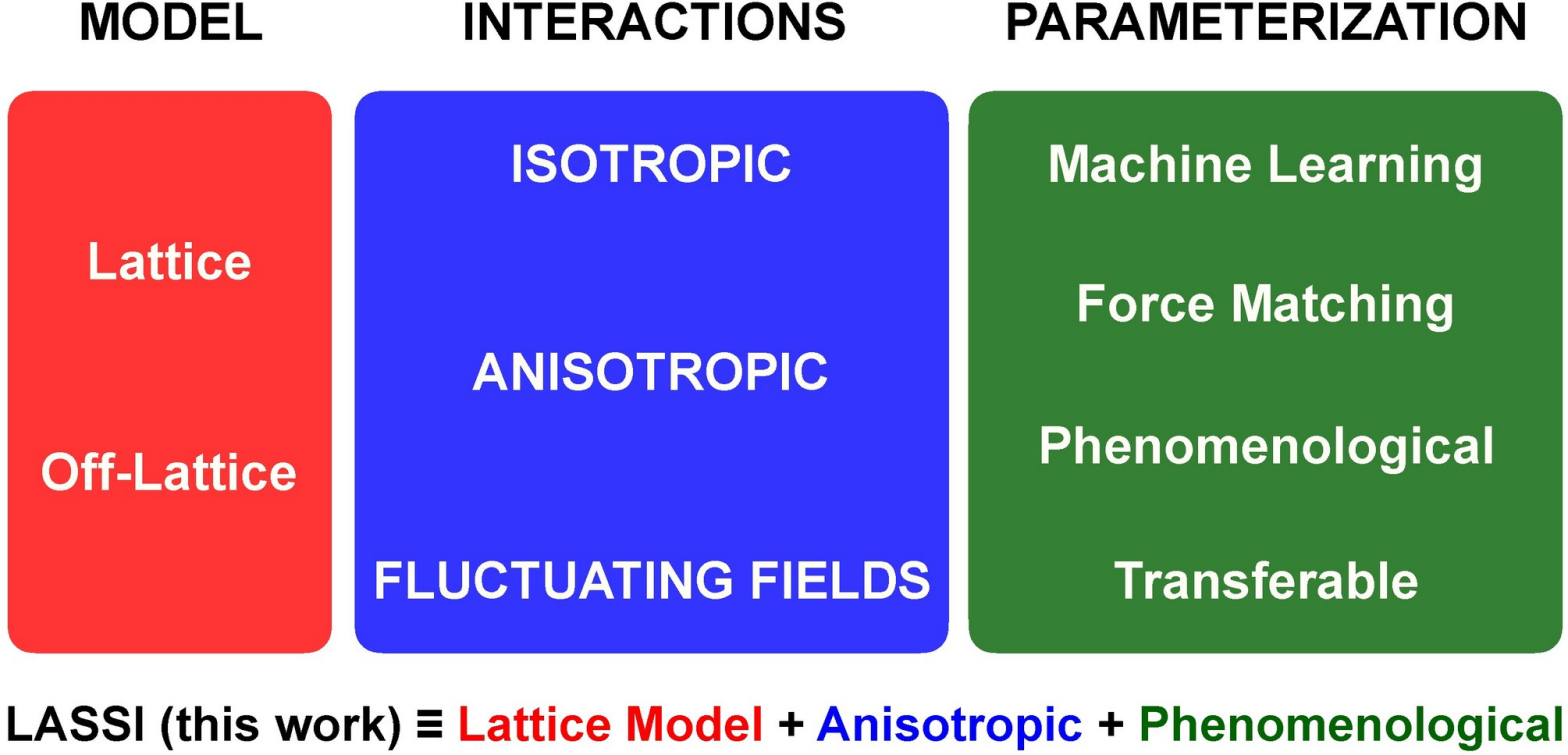
Considerations that go into designing a coarse-grained model. As discussed in the text, the choice of a coarse-grained model has at least three ingredients. These include the type of conformational space (lattice or off-lattice), the nature of the interactions among entities that are represented in the coarse-grained description (isotropic, anisotropic or fluctuating fields), and the parameterization approach. LASSI, as described here, is based on a lattice model that uses anisotropic interactions and a phenomenological model.

Coarse-graining, an essential aspect of making simulations of large numbers of multivalent proteins a tractable proposition, comes in different flavors [69]. For simplicity, we divide considerations that go into the development of a suitable coarse-grained model into three categories (**Fig 2**). These are (1) the type of model, (2) the types of interactions among the entities in the simulation, and (3) parameterization of the interaction potentials for the model of interest. Two distinct choices for the type of model are the choice between simulations being performed using *lattice models* versus *off-lattice models*. In either space, one or all of the molecules can be represented explicitly using architectures that represent coarse-grained mappings of the protein of interest. Next, the interactions among the units that make up each protein can be modeled as being *isotropic* or *anisotropic*. This is true of simulations where proteins of interest are modeled explicitly. In contrast, numerical instantiations of *field theoretic* models model can also be brought to bear where only a single chain is modeled explicitly [60, 70]. The remaining protein and solvent molecules are modeled as fields whose fluctuations are concentration dependent [71]. The effects of all other molecules influence the phase behavior of the explicitly modeled single chain through interactions of the chain with the field. Finally, the choice of interaction potentials is the bedrock of every simulation. The functional forms and parameters for potentials can be derived using *phenomenological* considerations intended to enable calculations of the “what if” variety–an approach that is common practice in statistical and polymer physics. One can also obtain system-specific parameters using information gleaned from atomistic simulations of smaller-scale facsimiles of the system of interest. These system-specific parameters are derivable using *force matching* methods pioneered by Voth and coworkers [72–77] or by prescribing a functional form for the potential that describes interactions in the coarse-grained space and employs *machine learning* methods to derive the relevant parameters [74, 78]. Finally, one can adopt approaches similar to the parameterization of molecular mechanics forcefields and develop a single *transferable* model that should be applicable to a large number of disparate systems.

Different coarse-grained simulations represent different combinations of model, interaction type, and parameterization. Two illustrative examples for deriving coarse-grained models for simulations of phase behavior of multivalent proteins come from the works of Ruff *et al*. [78] and Dignon *et al*. [64, 65]. Ruff et al. show how one can generate off-lattice models, of bespoke resolutions and learned parameters for isotropic potentials derived using machine learning that leverages information gleaned from atomistic simulations of individual proteins and protein oligomers. Dignon *et al*. also use an off-lattice model based on isotropic potentials whose parameters are designed to be transferable across disparate intrinsically disordered proteins.

It is worth emphasizing that at this juncture, there is no valid reason to stipulate that one combination of approaches for deriving a coarse-grained model is superior to another combination. As noted by Das *et al*. [67, 68], all models have distinct strengths and limitations. However, for specific applications, some methods afford quantifiable computational advantages over others. In our case, we are interested in uncovering conceptual nuances of phase diagrams for multicomponent systems that comprise of multivalent proteins characterized by anisotropic interactions among domains / motifs. As noted above, these systems can be mapped onto a stickers-and-spacers architecture. The questions we are interested in answering pertain to the order parameters that describe phase behavior, the impact of chain connectivity and spacer effective solvation volumes on phase behavior, and the determinants of the shapes of phase diagrams of multicomponent systems where phase transitions are driven by heterotypic as well as homotypic interactions. In this context, it is noteworthy that lattice models have been adapted to model phase transitions for systems comprising of different numbers of multivalent protein and RNA molecules [28–30, 43, 79–81].

In the present work, we provide a formal description of the design and implementation of system-specific lattice models for simulating phase transitions of multivalent proteins. The simulation engine, known as LASSI for **LA**ttice simulation engine for **S**ticker and **S**pacer **I**nteractions, formalizes the approaches that have been developed and deployed in recent studies [28–30, 79, 80]. Accordingly, LASSI combines a lattice model with anisotropic interactions among stickers and the model, at least in the current formalism, is derived based on phenomenological considerations (**Fig 2**). Ongoing work shows that a machine learning methodology known as CAMELOT [78] can be adapted for using LASSI as a tool to model sequence-specific phase behavior. We describe the design of LASSI, focusing first on the overall structure of the model, the Monte Carlo sampling, and their justification for generic multivalent proteins. We further describe the calculation of order parameters for quantifying phase separation and percolation. Then, using two specific examples of linear and branched multivalent protein systems, we illustrate the deployment of LASSI to two biologically relevant systems. In both systems, we make *a priori* assumptions regarding the identities of stickers and spacers, which is a requirement for the deployment of LASSI. Although we focus here on systems with a few components, it should be emphasized that the design of LASSI is able to handle a wide range of multicomponent systems.

## Materials and methods

Considerations that go into the development of a suitable lattice model include (a) the choice of the *mapping* between a specific multivalent protein of interest and a lattice representation, (b) the *parameterization* of the strengths and ranges of interactions for all unique pairs of beads and vacancies, (c) the *design* of move sets and acceptance criteria for Monte Carlo simulations that enable the sampling of local and collective motions of large numbers of lattice-instantiated multivalent proteins, (d) the efficient *titration* of key parameters such as protein concentrations and interaction strengths, and (e) the *extraction* of phase boundaries in terms of known and hidden collective parameters, which become the relevant *order parameters* for phase transitions of interest.

### Generating lattice representations of multivalent proteins

For a given linear or branched multivalent protein, we first choose a suitable mapping between the protein degrees of freedom and a lattice representation. The conformational space is a simple cubic lattice with periodic boundary conditions used to mimic a macroscopic system. Phase transitions represent the collective effects of large numbers of molecules, and simulations have to include at least 10^3^–10^4^ protein molecules to observe facsimiles of these collective transitions in finite sized systems [82]. Further, we need to be able to test for the effects of finite size artefacts and this requires a titration of the effects of varying the numbers of molecules. Accordingly, the lattice has to be large enough to accommodate at least 10^3^ molecules of each type for the most dilute concentrations. Often, we might need to increase the number of molecules to be of *O*(10^4^). Accordingly, a one-to-one mapping between the protein degrees of freedom and a lattice representation would lead to a computationally intractable model. Instead, we adopt system-specific coarse-graining approaches, whereby the coarse-graining is guided by *a priori* rigorous or phenomenological knowledge of the identities of *stickers* versus *spacers*. For disordered proteins, the stickers within disordered regions often correspond to single amino acid residues or short linear motifs. For multivalent folded proteins, the stickers are either an entire protein domain or sectors on domain surfaces [28, 29]. Residues corresponding to spacers may either be modeled explicitly, where one or more spacer residues are modeled by a single bead on the lattice site, or be modeled as phantom tethers, where the intrinsic lengths of tethers are calibrated in terms of the numbers of lattice sites [28, 29]. In both cases, the tethers can stretch, bend, and rotate and these degrees of freedom contribute to density inhomogeneities that are the result of altered patterns of inter-sticker interactions.

### LASSI and bond fluctuation models

The structure of LASSI is inspired by the bond fluctuation model (BFM) for lattice polymers [83]. This is a general lattice model for simulations designed to extract equilibrium conformational distributions and dynamical attributes of polymers in dilute solutions as well as dense melts. There are two versions of the BFM *viz*., the Carmesin-Kremer BFM or CK-BFM [84] and the Shaffer BFM or S-BFM [83]. Both models are based on the use of simple cubic lattices, which discretizes the conformational space for polymers.

In the CK-BFM [84], each repeating unit or monomer within a polymer is modeled as a 3-dimensional cube where the 8 corners of the cube occupy lattice sites and bond vectors connect pairs of monomers. Overlap of monomers is associated with an energetic penalty, and each bond vector can have up to 108 distinct directions. The choice of bond vector set encodes the geometry of the polymer and places constraints on the bond lengths and bond angles. All other interactions are governed by the inter-monomer potentials, and evolution of the system through conformational space is driven by changes to the overall potential energy. In contrast, the S-BFM places each monomer on a single lattice site. Covalently bonded monomers are connected by bonds that are constrained to be of three types, leading to chains that have bonds of length 1, $\sqrt{2}$ or $\sqrt{3}$ in units of lattice size. Monte Carlo moves with suitable acceptance criteria can be designed for both types of BFMs. The simulations are used to generate equilibrium conformational distributions of lattice polymers in either dilute or dense phases. The move sets control the overall polymer dynamics and the acceptance of different types of moves and the calculation of correlation functions allows one to compute dynamical quantities for lattice polymers [83]. If we were to use either of the established BFMs without modification, then each amino acid residue would be modeled as a monomer, and such an approach would be useful when the identities of stickers and spacers remain ambiguous. This approach underlies a different simulation engine known as PIMMS [43].

LASSI is a generalization of the S-BFM that also adapts features of the CK-BFM. Given a choice of the mapping for coarse-graining, each multivalent protein is described as a chain of non-overlapping monomers *viz*., beads that occupy sites on a 3-dimensional cubic lattice. Note that the choice of a single site per bead is similar to that of the S-BFM, although the bead, which is a sticker or spacer monomer, need not be the monomeric unit, *i*.*e*., an amino acid residue in the case of proteins. Each sticker monomer is linked to its adjacent sticker on the chain via either a phantom tether or a set of spacer beads that occupy individual lattice sites [28, 29]. A spacer / tether length of unity implies that adjacent monomers are within $\sqrt{3}$ lattice units of one another (**Fig 1D**). The choice of the spacer length will be sequence-specific or more precisely, specific to the architecture of the protein of interest.

Inter-monomer (sticker-sticker, sticker-spacer, and spacer-spacer) interactions are modeled as contact-based pairwise interactions. A sticker monomer can bind to another sticker monomer that occupies an adjacent lattice site with an interaction energy that depends on the types of both monomers. Monomers are considered to be adjacent to one another if they are within a lattice distance of $\sqrt{3}$. By this criterion, each lattice site occupied by a sticker monomer will have 26 adjacent lattice sites. This is reminiscent of the interaction geometry of a CK-BFM for each monomer. In the current implementation of LASSI, the interactions are mutually exclusive, implying that a sticker cannot interact simultaneously with more than one other sticker, even though there are 26 adjacent sites that the interaction partner can occupy. If the sticker in question is already engaged in another inter-monomer interaction with stickers or spacers, then the unoccupied sites of the sticker will be unavailable for interaction. The combination of the geometry of the interaction sites per monomer and the single occupancy constraint leads to anisotropic interactions between sticker interactions. This feature is unique to LASSI and it is not incorporated in other variants of BFMs; this allows us to deploy LASSI for modeling heteropolymeric systems. In the context of LASSI, we note that stickers are distinguished by their ability to participate in anisotropic or isotropic interactions. In contrast, explicitly modeled spacer sites only participate in isotropic interactions with other spacer or sticker sites. Furthermore, the interaction strengths involving spacers are typically weaker than those involving stickers. However, it is worth emphasizing that these distinctions only matter inasmuch as LASSI allows us to capture a numerical instantiation of the stickers-and-spacers model. For simplicity, one might simply think of LASSI as a model that has sites that are differentiated by whether or not they can involve themselves in anisotropic interactions, by their intrinsic site valence (a variable that we do not titrate in this work), and by the comparative magnitudes of site-site interaction strengths.

### Setup of simulations

A system with *n* multivalent proteins is in reality an *n*+1 component system since the solvent is the implicit component. In LASSI, sites that are not occupied by protein units automatically represent solvent sites. Although the interaction potentials do not explicitly include terms between solvent and protein sites, the effective interaction strengths between pairs of protein units represent an averaging over protein-protein, protein-solvent, and solvent-solvent interactions. The solvent sites, *i*.*e*., the sites that are not occupied by protein units, represent contributions from the solvent to the overall translational and mixing entropies. Simulations are initiated by randomizing the positions of protein units, subject to the constraints of chain connectivity.

The parameters that are set at the start of each LASSI simulation include the total number of molecules *n*_*i*_ of type *i* and the size of the lattice *L*, from which we can calculate the total number *n* of all protein components $n=\sum_{i}n_{i}$ and the concentration or number density of each protein *c*_*i*_ = *n*_*i*_/*L*^3^. The setup also includes stipulations for the architectures of each protein such as specification of the number of monomers per chain, the overall topology of each protein (linear *vs*. branched), the lengths of spacers, and the types of spacers (implicit / phantom *vs*. explicit) [28–30, 79, 80]. The number of monomers per molecule will equal the sum of the number of stickers and spacers if spacer residues are modeled explicitly. Alternatively, if spacers are modeled as phantom tethers, then the number of explicitly modeled monomers will equal the number of stickers. Specification of the energetics of the system includes specification of the simulation temperature in normalized units, homotypic and heterotypic interaction strengths between pairs of stickers, the energetic cost for the overlap of stickers, and the interaction strengths between sticker and spacer sites if the spacers are modeled explicitly.

### Design of monte carlo move sets

Our goal is to compute architecture-specific phase diagrams for systems comprising of one or more types of linear or branched multivalent proteins. This requires a simulation strategy that enables the sampling of the full spectrum of coexisting densities and networked states for multivalent proteins. Accordingly, the conformations of randomly initialized systems of proteins on a simple cubic lattice are sampled via a series of Markov Chain Monte Carlo (MCMC) moves that are designed to ensure efficient sampling of changes in protein density and networking while maintaining microscopic reversibility. We have developed and deployed a collection of moves and these are described below.

### Monte carlo sampling with biases

In LASSI, we have independent contributions from two main energetic sources. Monomer units are not allowed to overlap, and this can be described by a position-dependent energy *E*_pos_ where *E*_pos_ = 0 or ∞. On the other hand, inter-monomer pairwise interactions also contribute to the total energy, and *E*_rot_ denotes the sum over all of the effective pairwise inter-monomer interaction energies. The subscript “rot”(rotational) indicates the fact that for a pair of nearest neighbor stickers their interaction energies are actually governed by their mutual orientations. Accordingly, the total system energy in a specific configuration *i* is written as:

$$E_{i}=E_{i,\mathrm{pos}}+E_{i,\mathrm{rot}};$$ (1)

The equilibrium probability associated with configuration *i* is given by the Boltzmann distribution as:

$$p_{i}\propto \exp\left(-\beta E_{i}\right)=\exp\left(-\beta E_{i,\mathrm{pos}}\right)\exp\left(-\beta E_{i,\mathrm{rot}}\right);$$ (2)

In Eq (2), β is the inverse of the simulation temperature in units of the Boltzmann constant (effectively, *k*_*B*_ = 1 energy unit / temperature unit). The frequency with which a transition from configuration *i* to *j* is proposed will be governed by the elements *T*_*ij*_ of the targeting matrix **T**. The proposed transition is accepted / rejected based on the elements *A*_*ij*_ of the acceptance ratio matrix **A**. A MCMC move that transitions the system from configuration *i* to *j* defines a flow in configuration space and this flow has to satisfy microscopic reversibility:

$$T_{ij}A_{ij}p_{i}=T_{ji}A_{ji}p_{j};$$ (3)

If the targeting matrix is symmetric, then *T*_*ij*_ = *T*_*ji*_ and the acceptance ratios such as those prescribed by Metropolis *et al*. [85] will ensure the preservation of microscopic reversibility. However, if biases are incorporated into the targeting matrix, which is often essential to enhance the sampling of configurations that contribute to density inhomogeneities and the making / breaking of bonds in dense networks, then the elements of the acceptance ratio matrix have to be designed to ensure the preservation of microscopic reversibility. We deploy a general strategy of using biased moves to enhance the sampling of different mutual orientations among pairs of stickers. The incorporation of these orientational biases is accounted for by modifying the acceptance criterion of Metropolis *et al*. [85] whereby each element of **A** is written as:

$$\begin{array}{c} \left(\frac{A_{ij}}{A_{ji}}\right)=\left(\frac{T_{ji}p_{j}}{T_{ij}p_{i}}\right)\\ \mathrm{such}\;\mathrm{that:}A_{ij}=\min\left\{1,\frac{T_{ji}p_{j}}{T_{ij}p_{i}}\right\} \end{array};$$ (4)

For a symmetric targeting matrix, we recover the standard acceptance ratio of Metropolis *et al*. [85] *viz*.,

$$\left(\frac{A_{ij}}{A_{ji}}\right)=\left(\frac{p_{j}}{p_{i}}\right)\;\mathrm{or}\;A_{ij}=\min\left\{1,\frac{p_{j}}{p_{i}}\right\};$$ (5)

Since the moves within LASSI generally involve orientational biases, the elements *T*_*ij*_ are rewritten in terms of a Rosenbluth weighting factor *W*_*j*_ [86, 87] whereby:

$$T_{ij}=\frac{\exp\left(-\beta E_{j,\mathrm{rot}}\right)}{W_{j}};$$ (6)

Substituting (6) into (4) leads to:

$$A_{ij}=\min\left\{1,\frac{W_{j}}{W_{i}}\exp\left[-\beta \left(E_{j,\mathrm{pos}}-E_{i,\mathrm{pos}}\right)\right]\right\};$$ (7)

The specific form for the weighting factors *W*_*i*_ will depend on the type of move because the extent of asymmetry in the targeting matrix will depend on the nature of the bias incorporated into the biasing move that proposes a transition from *i* to *j*. The specific forms for weighting factors are discussed in the context of the move types that are introduced next.

### Rotational moves

A monomer is in an associated or a dissociated state and in the associated state it has a specified binding partner. This defines the rotational state of a monomer. To change the rotational state, we randomly pick a monomer from the system, and exhaustively sample all 26 adjacent sites to construct a list of potential binding partners. The rotational state of the monomer is changed, at random, by drawing a random integer *k* from a uniform distribution between [0, *b*], where *b* is the number possible binding partners available to the monomer. If *k* = 0, the monomer is set to be in a dissociated state. Otherwise, the *k*^th^ candidate bond (reversible physical crosslink) is formed and the state of the monomer is set to be in an associated state. If the monomer cannot be involved in a rotational interaction, as would be the case for an explicitly modeled spacer, the rotational move is rejected outright. The accessible volume for rotational interactions is within a cube of unit volume centered on the randomly chosen monomer (see **S1 Fig** for a 2-dimensional representation), and hence, each sticker monomer will have at most *b*_max_ = 3^3^–1 possible sites as neighbors. Since this number is not large, we sample all 26 possible interaction sites.

### Local moves

This move serves as the basic unit of local displacement of monomers–be they stickers or spacers. A randomly chosen monomer is moved from position **r**_*i*_ to **r**_*j*_ = **r**_*i*_ + Δ**r**. Acceptance of the move is predicated on the move not leading to an overlap with a site occupied by another monomer and the satisfaction of linker constraints. The choice for Δ**r** is made by uniformly sampling each component from the interval [–2,2] such that $\left|\Delta r\right|\leq 2\sqrt{3}$ (shown in **S2 Fig**) moves if the selected monomer is in the interior of a molecule [88], and they become analogous to *end rotation* moves if end monomers are selected [89].

Local moves have a rotational bias in LASSI and the Rosenbluth factor is calculated as follows. Starting with Eq (7), we shall designate the chosen monomer by index *k*. In configuration *i*, assume that monomer *k* has a binding partner of index *l*. Typically in coarse-grained systems there are a finite number of unique monomer types, and thus it is more efficient to simply define interaction energies between different monomer types than between all monomer pairs. The energy associated with the bond between monomers *k* and *l* is written as ε_*t*(*k*)*t*(*l*)_, where *t*(*x*) indicates the type of monomer *x*. The local move causes a change in binding partner, whereby the monomer *k* now binds to monomer *m*. The local move leads to a bond swap that causes a change in rotational energy, which is written as:

$$E_{i,\mathrm{rot}}-E_{j,\mathrm{rot}}=\varepsilon_{t\left(k\right)t\left(l\right)}-\varepsilon_{t\left(k\right)t\left(m\right)};$$ (8)

Use of Eq (8) in Eq (6) leads to:

$$\frac{T_{ij}}{T_{ji}}=\frac{\exp\left(-\beta \varepsilon_{t\left(k\right)t\left(l\right)}\right)W_{i;k}}{\exp\left(-\beta \varepsilon_{t\left(k\right)t\left(m\right)}\right)W_{j;k}};$$ (9)

In Eq (9), each Rosenbluth weight has an additional index in the subscript to indicate that the change in configuration is achieved by a change in the binding partner for the monomer *k*. To accelerate the creation of density inhomogeneities in supersaturated systems and facilitate the making and breaking of networks, we decompose *W*_*i;k*_ as:

$$W_{i;k}=W_{i;k}^{\left(a\right)}+W_{i;k}^{\left(d\right)};$$ (10)

The two terms in Eq (10) respectively represent the contributions to the Rosenbluth weights for monomers in associated (a) and dissociated states (d). First, we calculate the weight factors for the interacting monomers as a partition function over all nearest neighbor contributions, such that:

$$W_{i;k}^{\left(a\right)}=\sum_{l}\exp\left(-\beta \varepsilon_{t\left(k\right)t\left(l\right)}\right);$$ (11)

In Eq (11), the summation runs over all potential binding partners *l* (nearest neighbors) for the monomer *k*. To illustrate how the Rosenbluth factor is calculated, we assume that the system has only one type of interaction with the pairwise energy designated as ε. If the number of nearest neighbors for monomer *k* in configuration *i* is designated as *N*_*k;i*_, then the Rosenbluth weight factor in Eq (11) becomes:

$$W_{i;k}^{\left(a\right)}=N_{k;i}\exp\left(-\beta \varepsilon \right);$$ (12)

Setting $W_{i;k}^{\left(d\right)}=1$ to incorporate a bias towards associated states, and using the simplification that leads to Eq (12), we rewrite Eq (9) as a definition of the acceptance criterion for the move from configuration *i* to *j* via local move involving monomer *k* as:

$$A_{ij;k}=\min\left\{1,\frac{N_{j;k}+1}{N_{i;k}+1}\exp\left[-\beta \left(E_{j,\mathrm{pos}}-E_{i,\mathrm{pos}}\right)\right]\right\};$$ (13)

This choice for the acceptance criterion ensures that detailed balance is preserved while enhancing the sampling of configurations characterized by the breaking of old bonds and the forming of new ones.

### Reptation–or slithering snake–moves

In dense configurations, it becomes difficult to realize large-scale translational or rotational motions of polymers. The *slithering snake move* is a Monte Carlo instantiation of reptation as first conceived by de Gennes [90]. In this move, a chain is chosen at random, and the monomer at one end of the target chain is moved to a new position. The remaining monomers within the target chain are then successively moved such that monomer *m* along the chain moves into the previous position of monomer *m*–1 (**S3 Fig**). This move relies on an inherent symmetry of chain molecules, because bond lengths between monomers are the same; if one swaps monomers across chains, the identity of the chain remains invariant. However, this move cannot be used if the molecule has heterogeneous bond lengths or if it is a branched polymer.

The reptation move is rotationally biased, and this is true for every monomer in a chain. The bias is independent for each monomer and accordingly, the Rosenbluth factor for a single reptation move can be calculated from the Rosenbluth factors for each monomer-specific local move. In configuration *i* we obtain:

$$W_{i}=\prod_{m}W_{i;m}=\prod_{m}\left(N_{i;m}+1\right);$$ (14)

In Eq (14), the product runs over all monomers *m* within the chain of interest. The acceptance criterion for a reptation move takes the form:

$$A_{ij}=\min\left\{1,\frac{\prod_{m}\left(N_{j,m}+1\right)}{\prod_{m}\left(N_{i,m}+1\right)}\right\}\exp\left[-\beta \left(E_{j,\mathrm{pos}}-E_{i,\mathrm{pos}}\right)\right];$$ (15)

The inclusion of the bias for every interacting monomer, rather than just the end monomers, is to emulate how a real transiently bonded polymer would slither along its contours. Note that for strict detailed balance, the Rosenbluth factors for the two end monomers should be calculated and added to the acceptance criterion, but the current implementation of LASSI uses the first trial position that satisfies the position constraints.

### Double pivot moves

These moves swap a part of a chain with the corresponding part of another chain of the same type. A monomer is picked at random; it is denoted as *i*_*m*_, where *i* is the monomer index within a chain, and *m* is the chain index. A search is then performed around the monomer within a prescribed distance for a monomer within the same type of chain. The requirement for the search is that the monomer of interest be one index ahead along its own chain, (*i*+1)_*n*_. Next a check is performed to ensure that the distance between *i*_*m*_ and (*i*+1)_*n*_ is within the bond constraint connecting *i*_*m*_ to (*i*+1)_*m*_, and that the distance between (*i*+1)_*m*_ and *i*_*n*_ is within the bond constraint for *i*_*m*_ and (*i*+1)_*m*_. Each monomer (*i*+1)_*n*_ that satisfies each of these constraints is stored and one of these is randomly picked for the double pivot move (**S4 Fig**). The move is always accepted if there is a candidate because only connectivity changes unless bonds are modeled using elastic springs.

The purpose of this move is to engender large configurational changes in dense polymer melts, which approximate the dense phases formed upon phase separation. In dense regions the rate of acceptance of local moves decrease precipitously. At high enough densities, polymers become entangled and local moves reduce to slithering-snake moves and polymers are restricted to motions along tubes around one another [91, 92]. Therefore, rather than physically moving polymers to create a change in configurations, we incorporate move sets that break and make bonds while ensuring that monomers do not overlap, and that bond constraints are always satisfied. If two chains are close enough to each other that the bonds between two monomers can be swapped, then such the double pivot move results in a large configurational change for both chains, and for the system.

### Chain and cluster translation moves

The chain translation move is designed to move single chains while forming new bonds at the proposed location. This move attempts to translate the center of mass of a chain *i* from **r**_*i*_ to **r**_*j*_ = **r**_*i*_ + Δ**r** where $\left|\Delta r\right|\leq \frac{\sqrt{3}L}{4}$ and *L* is the size of the simulation cell. Multiple trial displacements are proposed until a trial position that does not result in steric clashes for the entire molecule is generated. The move is then attempted. As with the slithering snake move, each monomer in the molecule that is translated will have an orientational bias. Accordingly, the Rosenbluth factors are calculated as in Eqs (14) and (15). The translational move results in large displacements for single chains and correctly biases the system for efficient sampling of configurations with alternate interaction patterns.

Translational moves can also be applied to clusters of molecules. A connected cluster refers to a collection of unique chains connected via rotational interactions. A proposed move only results in a translation, and the move is readily accepted if there is no steric clash. Since no new physical bonds are created at the proposed location, the cluster remains invariant and the move is accepted. Naively this move might seem unnecessary as this move simply moves clusters around. However, once a physical bond has formed between two molecules, it is highly unlikely for any of the non-cluster translation moves to move the centers-of-masses of clusters closer together.

### Considerations for setting move set frequencies

The structure of each move set serves as a guide for selecting an optimal set of frequencies. This leads to a set of heuristics that are as follows: (i) in the cluster move we pick a random chain from the system, perform a networking analysis on that chain, and then propose a displacement of the cluster. As the cluster size grows it is more likely that a randomly picked chain will be part of the largest cluster which itself will result in a steric clash after the proposed move. Therefore, the frequency of the cluster move should be low, if not the lowest, in the entire set. (ii) In the translational move, we pick a random chain from the system for translation; as the size of the largest cluster increases it becomes less likely for a proposed translation move to be accepted. However, unlike the cluster move the translation move is rotationally biased and thus results in new interactions being formed. Hence, translational moves enable single-molecule to cluster-surface interactions. Therefore, this move should be proposed more often than the cluster move, although not as often as rotational or local moves. (iii) The rotation move is computationally inexpensive and it enables the switching of physical bonds and should thus be proposed fairly frequently. (iv) Similarly, local moves and slithering snake moves are also rotationally biased, and they help with the local rearrangements of physical bonds. Local moves are the primary route to enable local conformational changes, and to enable local physical bond rearrangements. Therefore, local moves should be proposed most frequently. The slithering snake move is particularly effective because it allows for large local physical bond rearrangements in dense configurations. Thus, this move should also be proposed frequently, less so than local moves but more so than translation moves. Note that in a system where some molecules are non-linear or have heterogeneous linker lengths, the frequency would need to be higher since the move is rejected if an incorrect molecule is picked at random. (v) The double pivot move allows for large-scale changes to conformations within dense configurations and accordingly, this move should be proposed more frequently than both cluster and translation moves. One can track the acceptance ratios of each move over a very rough initial sweep across the relevant system parameters. Moves that are always rejected do not enable any changes in configuration and only add computational costs. Therefore, the frequency for that particular move should be lowered. This is especially the case for the cluster move in high-density systems.

### Identifying phase boundaries using measures for density inhomogeneities

In order to detect the onset of phase separation, we can calculate excess chemical potentials using the Widom particle insertion method [93] and equalize these chemical potentials across distinct phases. This process requires *a priori* knowledge of the densities of both phases. An efficient variant of this approach, based on fast Fourier transforms, was recently developed and deployed by Qin and Zhou [61]. They demonstrated their method for calculations of liquid-liquid coexistence curves for a patchy colloid model of γII-cyrstallin. Given that LASSI simulations are lattice-based, we instead rely on properties of pair distribution functions that help us diagnose the onset of phase separation and compute phase boundaries. Pair distribution functions are helpful because phase separation is the result of the system partitioning into phases of different densities. The pair distribution, which is a reduced-dimension partition function, serves as a rigorous thermodynamic and structural measure of the average local density and inhomogeneities of density. To first order, the density fluctuations are quantified by averaging over all orientations. Accordingly, the pair distribution function can be converted to a radial distribution function that allows us to probe local densities and local structural organization of molecules around one another. However, normalization of the pair distribution function requires some caution. The system contains polymer molecules and using a prior distribution that assumes an ideal gas of the chain monomers to normalize the pair distribution function is problematic because it does not accurately capture the effects of non-idealities due to chain connectivity. We leverage the efficient sampling of polymer fluids in LASSI and obtain suitable prior distributions by simulating the system of interest in the absence of sticker-sticker interactions.

**Fig 3:**
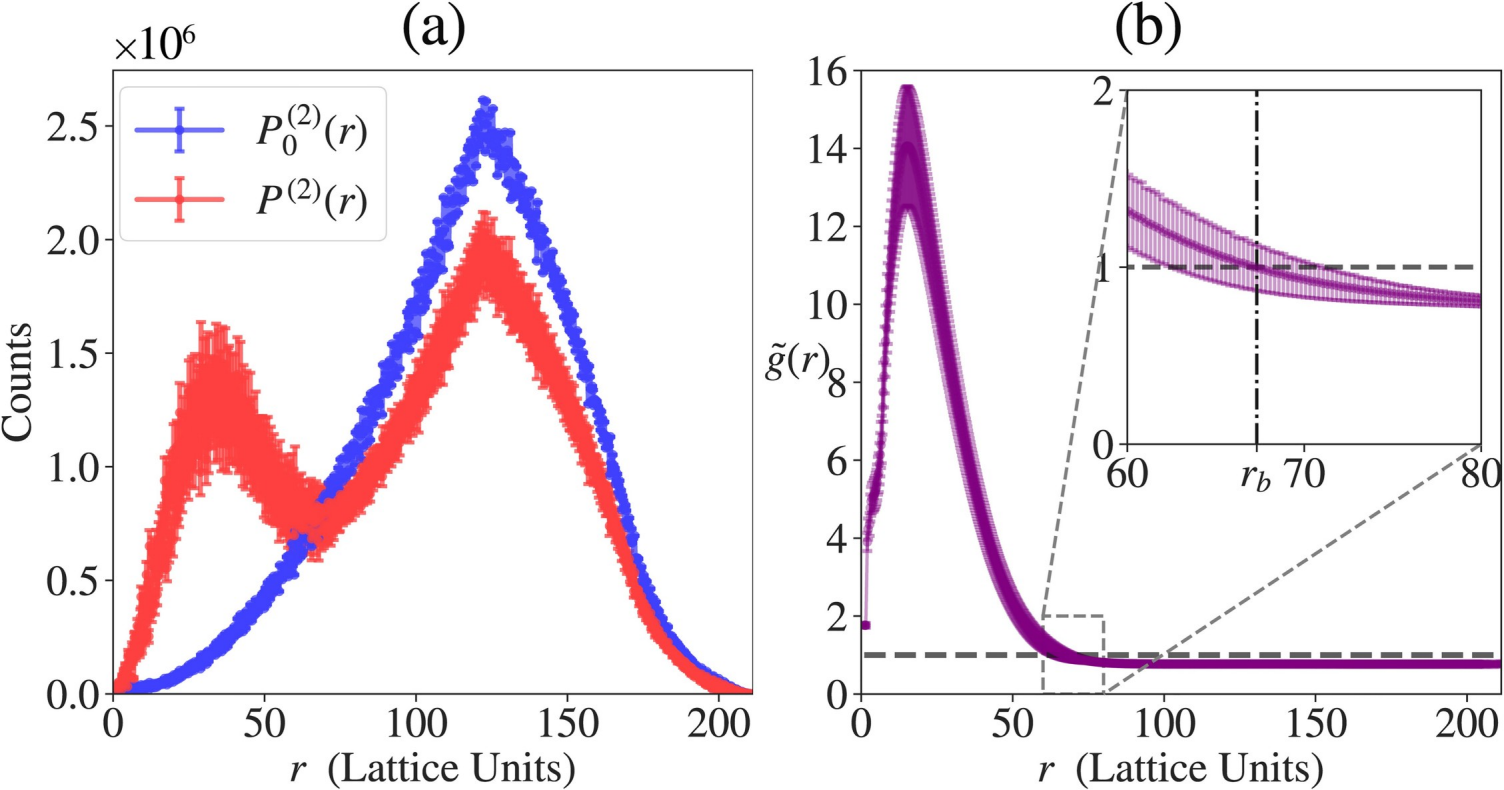
Distribution functions used for calculation of density inhomogeneity. The data shown are obtained from 5 independent simulations for the A_*n*_-B_*n*_ system with total protein concentration *c* = 6.89×10^−5^ voxels^-1^ and reduced temperature T* = 0.383. Error bars indicate standard deviations. (a) Pair distribution functions *P*^(2)^(*r*) and *P*_0_^(2)^(*r*), where the former is from the interacting system and the latter from the non-interacting system with chain connectivity (*prior pair distribution function*). Note that *P*^(2)^(*r*) shows two peaks, the first of which indicates dense phase formation. (b) Radial distribution function $\tilde{g}\left(r\right)$. This captures the droplet formation by a sharp and broad peak in the beginning. The inset shows *r*_b_ where $\tilde{g}\left(r\right)$ intersects the line corresponding to $\tilde{g}\left(r\right)=1$ line, delineating between the dense and solution phases. The global density inhomogeneity measure, $\bar{\rho }$, is obtained by integration of absolute deviation of $\tilde{g}\left(r\right)$ from 1.

The pair distribution function *P*^(2)^(*r*) quantifies the equilibrium distribution of distances between chain monomers, where *r* is the inter-monomer distance. If $P_{0}^{\left(2\right)}\left(r\right)$ denotes the prior pair distribution function calculated from simulations where the inter-sticker interactions are ignored (see **Fig 3A**), then the normalized radial distribution function is written as:

$$\tilde{g}\left(r\right)=\frac{P^{\left(2\right)}\left(r\right)}{P_{0}^{\left(2\right)}\left(r\right)}$$ (16)

The function $\tilde{g}\left(r\right)$ is a direct measure of the local density of the protein of interest. Since LASSI uses periodic boundary conditions, the maximal inter-monomer distance is $\frac{\sqrt{3}L}{2}$. Given this normalized $\tilde{g}\left(r\right)$, we note that if the system has short-range ordering as in a canonical liquid, the radial distribution function will oscillate around unity but eventually approach one as *r* → ∞. Conversely, if the system undergoes a density transition, $\tilde{g}\left(r\right)$ will have two distinct spatial regimes (**Fig 3B**): for small *r*, $\tilde{g}\left(r\right)$ will be characterized by a tall and broad peak such that $\tilde{g}\left(r\right)>1$ until $\tilde{g}\left(r\right)$ intersects the $\tilde{g}\left(r\right)=1$ line; this region corresponds to the dense phase and we shall denote the value of *r* at this intersection to be *r* = *r*_b_. For *r* > *r*_b_, $\tilde{g}\left(r\right)$ will be between 0 and 1, and for lattices that are large enough to avoid finite size artefacts, $\tilde{g}\left(r\right)$ will converge to a value lower than one and this corresponds to the density in the dilute phase region. Furthermore, $\tilde{g}\left(r\right)$ can be used to estimate the densities within the dense and dilute phases.

To quantify the global density inhomogeneity we introduce a simple measure, $\bar{\rho }$, which is calculated as follows:

$$\bar{\rho }=\left(\frac{1}{L}\right)\int_{0}^{\frac{\sqrt{3}L}{2}}\left|\tilde{g}\left(r\right)-1\right|V\left(\frac{r}{L}\right)dr$$ (17)

In Eq (17), *V*(*x*), the volume element for the normalized radial distance *x = r/L* defined as:

$$V\left(x\right)=\{\begin{array}{c} \begin{array}{cc} 4\pi x^{2}, & \mathrm{if}\;0<x\leq \frac{1}{2} \end{array}\\ \begin{array}{cc} 2\pi x\left(3-4x\right), & \mathrm{if}\;\frac{1}{2}<x\leq \frac{\sqrt{2}}{2} \end{array}\\ \begin{array}{cc} 2x\left(3\pi -12f_{1}\left(x\right)+f_{2}\left(x\right)\right), & \mathrm{if}\;\frac{\sqrt{2}}{2}<x\leq \frac{\sqrt{3}}{2} \end{array} \end{array}$$ (18)

$$\mathrm{and}\;\begin{array}{c} f_{1}\left(x\right)=\arctan\left(\sqrt{4x^{2}-1}\right),\\ f_{2}\left(x\right)=8x\arctan\left(\frac{2x\left(4x^{2}-3\right)}{\sqrt{4x^{2}-2}\left(4x^{2}+1\right)}\right) \end{array}$$ (19)

If $\bar{\rho }$ ≈ 0, the global density inhomogeneity in the system is small and this will be characteristic of a single homogeneous phase dominating the simulation volume. As $\bar{\rho }$ increases beyond zero, the system accommodates density inhomogeneities. We construct coexistence curves using a cutoff value of $\bar{\rho }$ = 0.025, which is universal to all systems, to delineate between a homogeneous system and one that has undergone phase separation.

### Quantitative assessments of finite size effects

The pair distribution function is central to our calculation of density inhomogeneities and constructing coexistence curves for a system of multivalent proteins simulated using LASSI. At the start of this section we emphasized the importance of including 10^3^–10^4^ distinct molecules within the simulation cell in order to avoid finite size artefacts. Prior to presenting detailed results that mimic specific systems, we present an analysis of finite size effects that we will confront if the requisite numbers of molecules are not included in the simulations. The data we present are for simulations of mimics of the protein FUS, specifically the A_n_ + B_n_ system introduced in the results section. The phenomenological mapping of this protein architecture onto a cubic lattice is discussed at the start of the results section. Here, we present an analysis that makes a crucial technical point about finite size effects.

**Fig 4:**
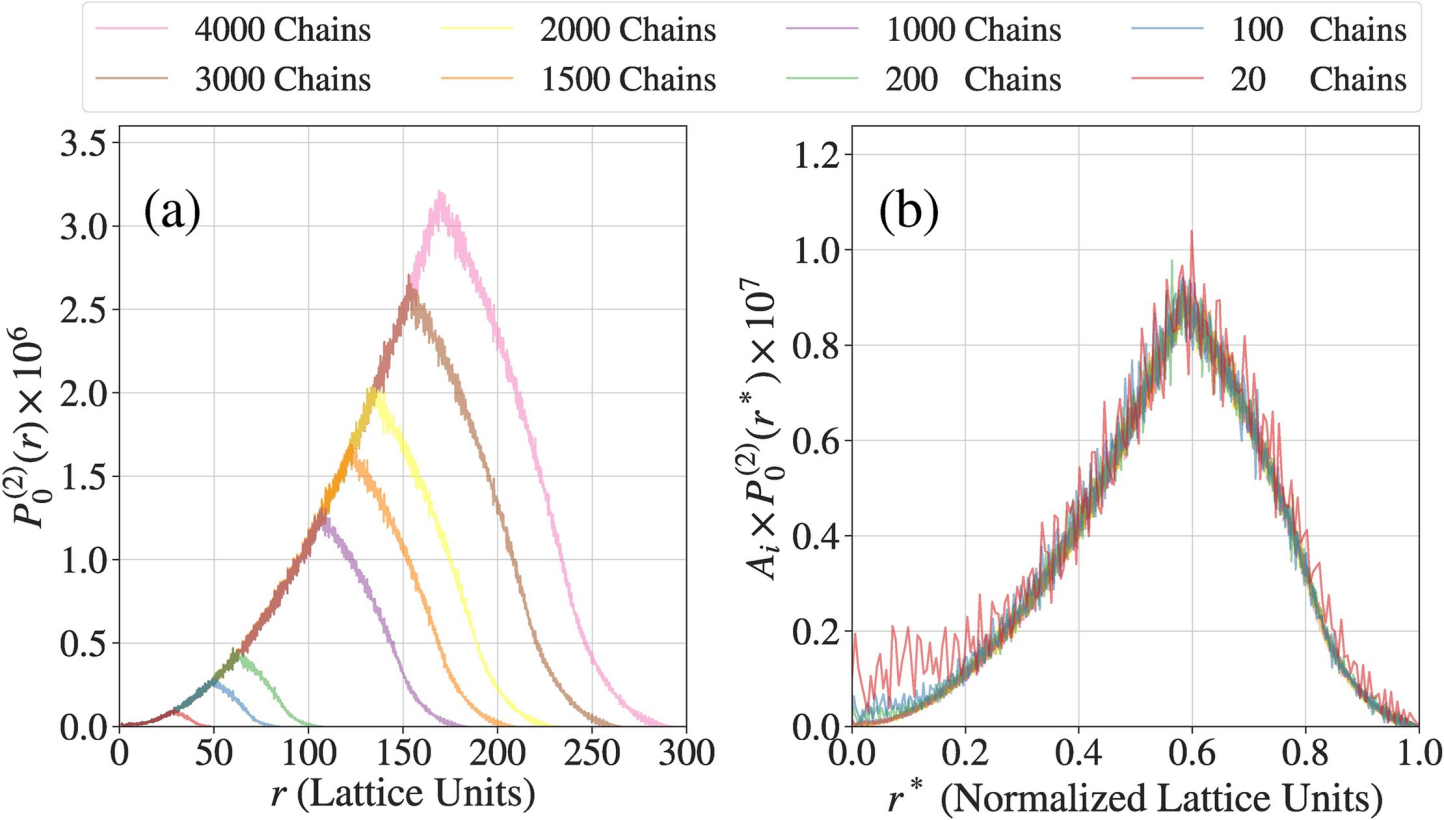
Assessment of finite size effects in simulations of ideal, non-interacting chains. (a) Pair distribution functions computed in terms of the spatial separation between chain units. The distributions are maximal at *r* = *L*/2, where *L* is the size of the simulation cell for a given system. Note that *L* increases as the number of polymers in the system increases. With the exception of the smaller systems, the ideal chains show self-similar behavior for different system sizes. (b) The data plotted in panel (a) are re-plotted in terms of the scaled variable $r*=\frac{2r}{\sqrt{3}L_{i}}$ where *L*_*i*_ is the size of the simulation cell for boxes with *i* molecules.

First, we start with simulations for ideal polymers. The data shown in **Fig 4** plots the pair distribution function $P_{0}^{\left(2\right)}\left(r\right)$ extracted for simulations of ideal models of FUS-like proteins. Results are shown for simulations that use 20 chain molecules of A_n_ + B_n_ as an example of a small system. These results are compared to those from simulations with 100, 200, 1000, 1500, 2000, 3000, and 4000 A_n_ + B_n_ molecules, respectively. The pair distribution functions have a self-similar character and this is revealed by plotting $P_{0}^{\left(2\right)}\left(r*\right)$ for all of the simulations, where r* is the reduced distance that accounts for the fact that for a similar concentration, the simulation cells are made larger (higher values of *L*, which is box size) as the numbers of molecules increase. This analysis shows that even for a truly ideal system, the smallest simulation comprising of only 20 molecules will generate noisy estimates of the pair distribution function. This clearly demonstrates the problems inherent to small systems where finite size effects are accentuated. Interestingly, for the ideal chain system, all simulations with 100 or more molecules yield similar pair distribution functions as assessed in **Fig 4B**.

**Fig 5:**
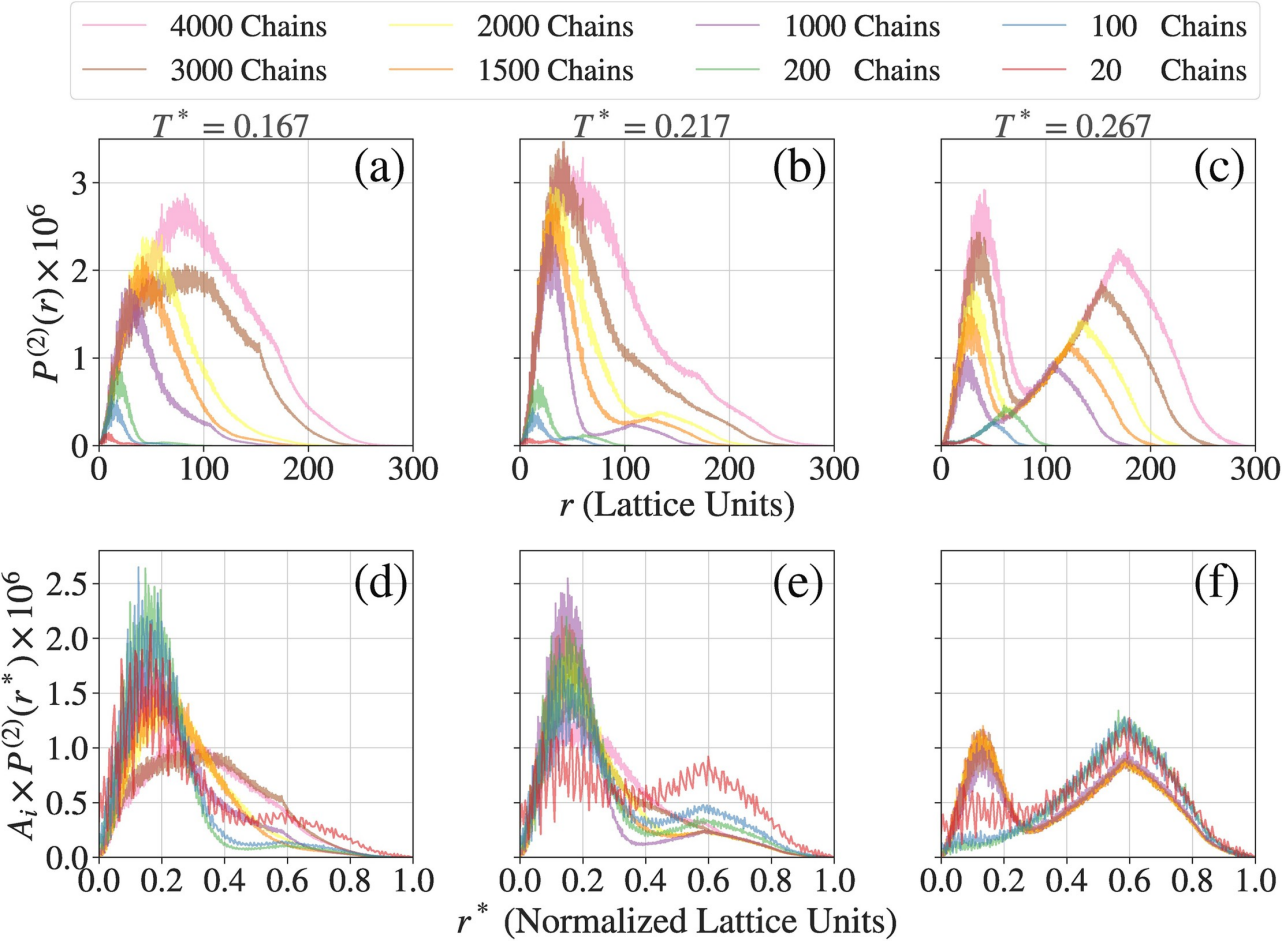
Assessment of finite size effects in simulations with real interacting chains. Panels (a), (b), and (c), respectively are the real chain equivalents of panel (a) in **Fig 4** computed for three different simulation temperatures that represent three different quench depths of the system into its two-phase regime. Panels (d), (e), and (f), respectively are the real chain equivalents of panel (b) in **Fig 9** computed for three different simulation temperatures that represent three different quench depths of the system into its two-phase regime.

Next we assessed the impact of finite size effects with all of the terms in the potential being included in the simulation. There are three columns, one each for different values of the reduced temperature T*, in **Fig 5**. As discussed in the results section, these values of T* place the system of interest in the two-phase regime, with the quench depth into the two-phase regime increasing as T* increases. The first row (a to c) of **Fig 5** shows the same data as **Fig 4A** while the second row (d to f) in **Fig 5** show the normalized data like **Fig 4B**. Each panel shows eight unnormalized pair distribution functions, one each for the systems with 20, 100, 200, 1000, 1500, 2000, 3000, and 4000 molecules, respectively.

The onset of phase separation should be manifest by the presence of a trough located between two peaks in the profiles for *P*^(2)^(r*). This is evident for T* = 0.267 (**Fig 5**, panel (c)) for all systems providing the numbers of molecules are greater than or equal to 10^3^. This qualitative trend is preserved even for T* = 0.217, although sampling difficulties in large systems become obvious in the noisy estimates for *P*^(2)^(r*). At the reduced temperature of T* = 0.167 we confront two problems: The small systems where the numbers of molecules are less than 10^3^ cannot support the distinction between a proper dense phase that coexists with a dilute phase. This behavior is similar to that observed for lower quench depths *i*.*e*., higher simulation temperatures as shown in panels (b) and (c) of **Fig 5**. However, as the system size grows, an additional problem arises and this has to do with large clusters becoming frozen, and thus inhibiting the achievement of equilibrium. This is evident from the pair distribution functions shown in panels (a) and (d) of **Fig 5** for systems where the numbers of molecules exceed 10^3^. To overcome this broken ergodicity and obtain reliable converged pair distribution functions, we need additional biasing potentials and temperature sweep approaches used recently [43] to break up frozen clusters and enable their coalescence. In the results that we report here, we use the system size titration to identify the reduced temperatures below which broken ergodicity becomes evident. We do not include data from these simulations in our constructions of phase diagrams. Importantly, our analysis confirms the presence of finite size effects for small systems and sets a lower bound on the numbers of molecules that are needed to observe facsimiles of phase separation as diagnosed by the calculated pair distribution functions. The conclusions drawn from analysis of the pair distribution functions are reinforced in our analysis of the radial distribution function $\tilde{g}\left(r*\right)$ shown in **S5 Fig**.

### Estimating the percolation transition line that delineates percolated and non-percolated networks

Associative polymers form networks characterized by physical crosslinks among stickers. Accordingly, we use the concept of a *cluster*, *viz*., a collection of unique chains connected via rotational interactions, to define the extent of percolation. In polymer melt simulations, the extent of percolation, known as the *gel fraction* in the polymer literature, is defined as the fraction of polymers participating in a percolating network that spans the simulation box in at least one direction [94]. More generally, we can use the fraction of polymers that make up the single largest cluster to quantify the onset of percolation and the changes to the extent of networking beyond the percolation threshold [95]. A molecular network cannot percolate the whole simulation cell when dilute and dense phases coexist. Accordingly, we choose the second definition for the order parameter that describes the percolation transition, and we denote this as ϕ_c_ [29].

Semenov and Rubinstein demonstrated that a percolation transition is purely a connectivity transition [45]. This implies that the identification of the percolation threshold is not achievable using a *bona fide* order parameter but instead relies on a suitable topological description. Here, we employ the midpoint of the ϕ_c_ vs. concentration curve to assess the onset of percolation and the percolation line or curve is obtained as the locus of points in the phase diagram for which ϕ_c_ = 0.5. In a system where finite size effects are minimized, the percolation transition is sharp having either a hyperbolic or sigmoidal shape as a function of concentration. Accordingly, the location of the percolation line will be relatively robust to the choice one makes for the percolation threshold.

## Results

We demonstrate the use of LASSI by applying it to study two archetypal systems that are known to undergo phase separation [24, 31, 33]. The systems are instantiations of linear and branched multivalent protein systems, respectively. The simulation results obtained for linear multivalent proteins illustrate how phase diagrams are generated when protein concentration (at a fixed stoichiometry) and temperature are the independent variables. In the second example that includes a branched multivalent protein and a linear peptide, the temperature is fixed, and the concentrations of the individual components are varied. The simulation parameters for both systems are summarized in **Table 1**. For each system, we conducted 5 independent simulations, each of which consists of 5×10^8^ MC steps after 5×10^6^ equilibration steps. The data were taken over the last half of the trajectories at a frequency of 5×10^5^ steps.

**Table 1:** Simulation parameters for system description.

	FUS-like system (see Fig 6)	N130 + rpL5 system (see Fig 10)
Bead notations	A/B: stickers N: neutral spacers	A/B: stickers N: neutral hub spacers
Number of stickers *s*_*i*_	*s*_A_ = 5, *s*_B_ = 5	*s*_A_ = 10, *s*_B_ = 5
Linker length *l*_*ij*_ (in lattice units)	*l*_AN_ = 1, *l*_BN_ = 1, *l*_NN_ = 4	*l*_NA_ = 1, *l*_AA_ = 3, *l*_BB_ = 3
Position-dependent energy *E*_pos_(**r**_1_, **r**_2_)	∞, if **r**_1_ = **r**_2_ 0, otherwise	∞, if **r**_1_ = **r**_2_ 0, otherwise
Pairwise interaction energy ε_*ij*_ (in temperature units)	ε_AB_ = -3, ε_*ii*_ = 0, ε_*i*N_ = 0	ε_AB_ = -3, ε_*ii*_ = 0, ε_*i*N_ = 0

### A FUS-like system as an example of a linear multivalent protein

Wang *et al*. [24] recently uncovered the molecular grammar that contributes to the driving forces for phase separation of the protein FUS and a family of related proteins. They showed that, to first order, *c*_sat_ ∝(*s*_Y_*s*_R_)^−1^, where *c*_sat_ is the measured saturation concentration of the FUS family proteins and *s*_Y_ and *s*_R_ are the number of tyrosine (Tyr) and arginine (Arg) residues, respectively. In FUS and other proteins of similar architectures, the Tyr residues are located primarily within the N-terminal disordered prion-like domain (PLD), whereas the Arg residues are located primarily within the partially disordered C-terminal RNA binding domain (RBD).

**Fig 6:**
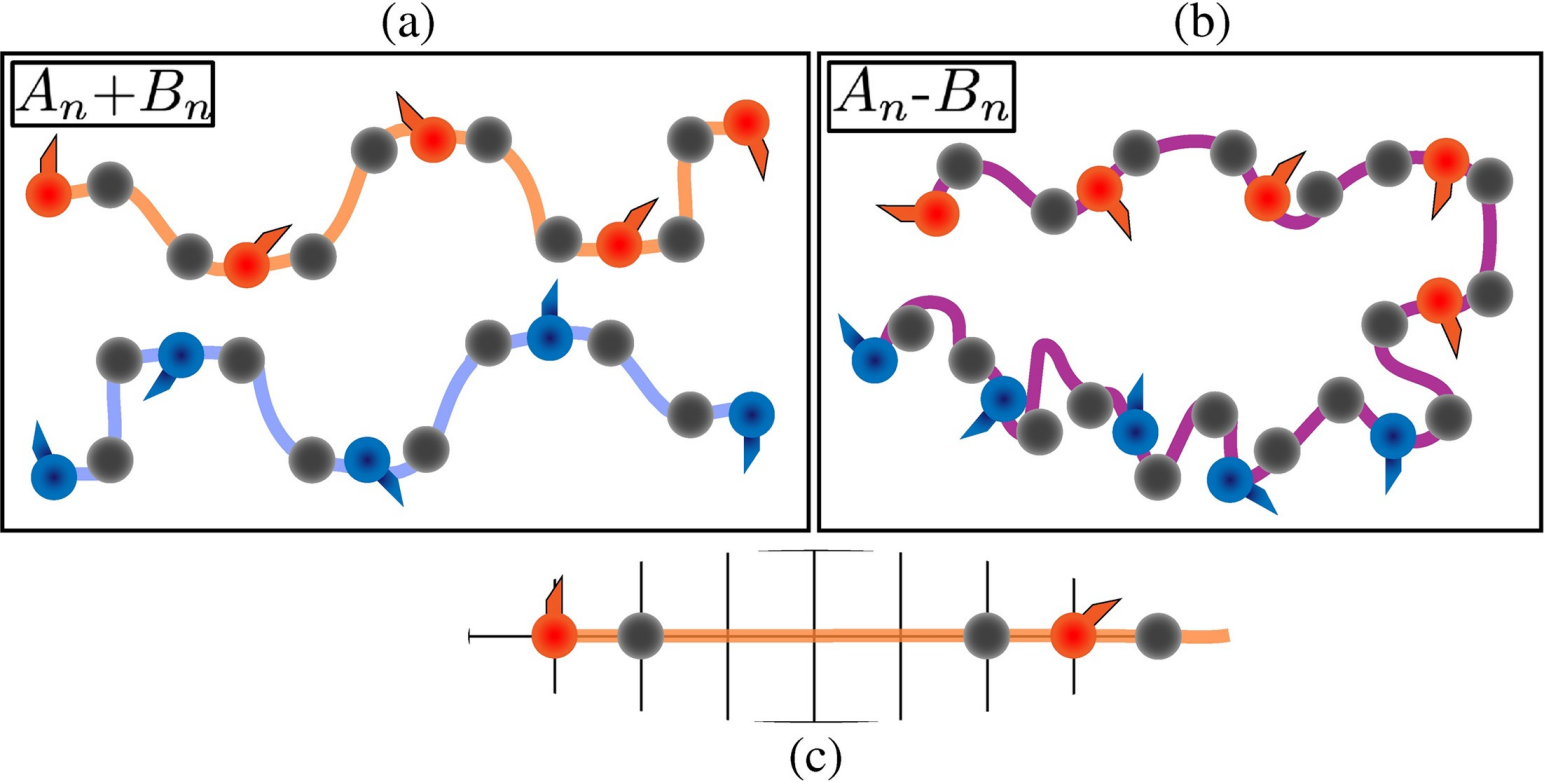
Architecture of the linear multivalent systems. (a, b) Cartoons to depict the A_*n*_+B_*n*_ and A_*n*_-B_*n*_ systems, respectively. Different colors of beads denote different species of stickers. Note that A_*n*_-B_*n*_ can be simply considered as A_*n*_+B_*n*_ where the two different sections of the proteins are joined together. (c) Linker lengths involved in the architecture (see also **Table 1**). Each sticker has a neighboring spacer bead that is 1 lattice site apart whereas the neighboring spacer beads are 4 lattice sites apart. This means that consecutive stickers are 6 lattice sites apart and also that the linkers connecting the two have a positive effective solvation volume.

Using mutagenesis experiments, Wang *et al*. established that Tyr and Arg residues are the stickers in the FUS family proteins. Accordingly, the zeroth-order stickers and spacers representation used to model FUS in LASSI comprises of two parts: An N-terminal mimic of the PLD encompassing Tyr residues as stickers and a C-terminal mimic of the RBD that encompasses Arg residues as stickers. Wang *et al*. also measured *c*_sat_ for a 1:1 mixture of independent PLDs and RBDs interacting in *trans*. The *c*_sat_ for this system is approximately twice that of the *c*_sat_ for full-length FUS. Given the block copolymeric architecture of FUS, we denote the PLD and RBD as A_n_ and B_n_, respectively for A and B-blocks of valence n. The model system of PLDs and RBDs interacting in *trans* is denoted as A_n_+B_n_ (**Fig 6A**), whereas the system mimicking full-length FUS where the stickers can interact in *cis* and in *trans* is denoted as A_n_-B_n_ (**Fig 6B**).

Within A_n_ and B_n_ blocks, spacers provide a uniform spacing of six lattice sites between stickers along the chain. We model spacers using a hybrid approach whereby a neutral spacer monomer is attached to each sticker with spacing of a single lattice site (**Fig 6C**). This choice was made to provide a distinction between A_n_-B_n_ and A_n_+B_n_. Accordingly, the relative concentration of neutral beads will be higher in A_n_-B_n_ when compared to A_n_+B_n_. This allows us to study linker-mediated differences between the driving forces for phase separation for A_n_-B_n_ vs. A_n_+B_n_

**Fig 7:**
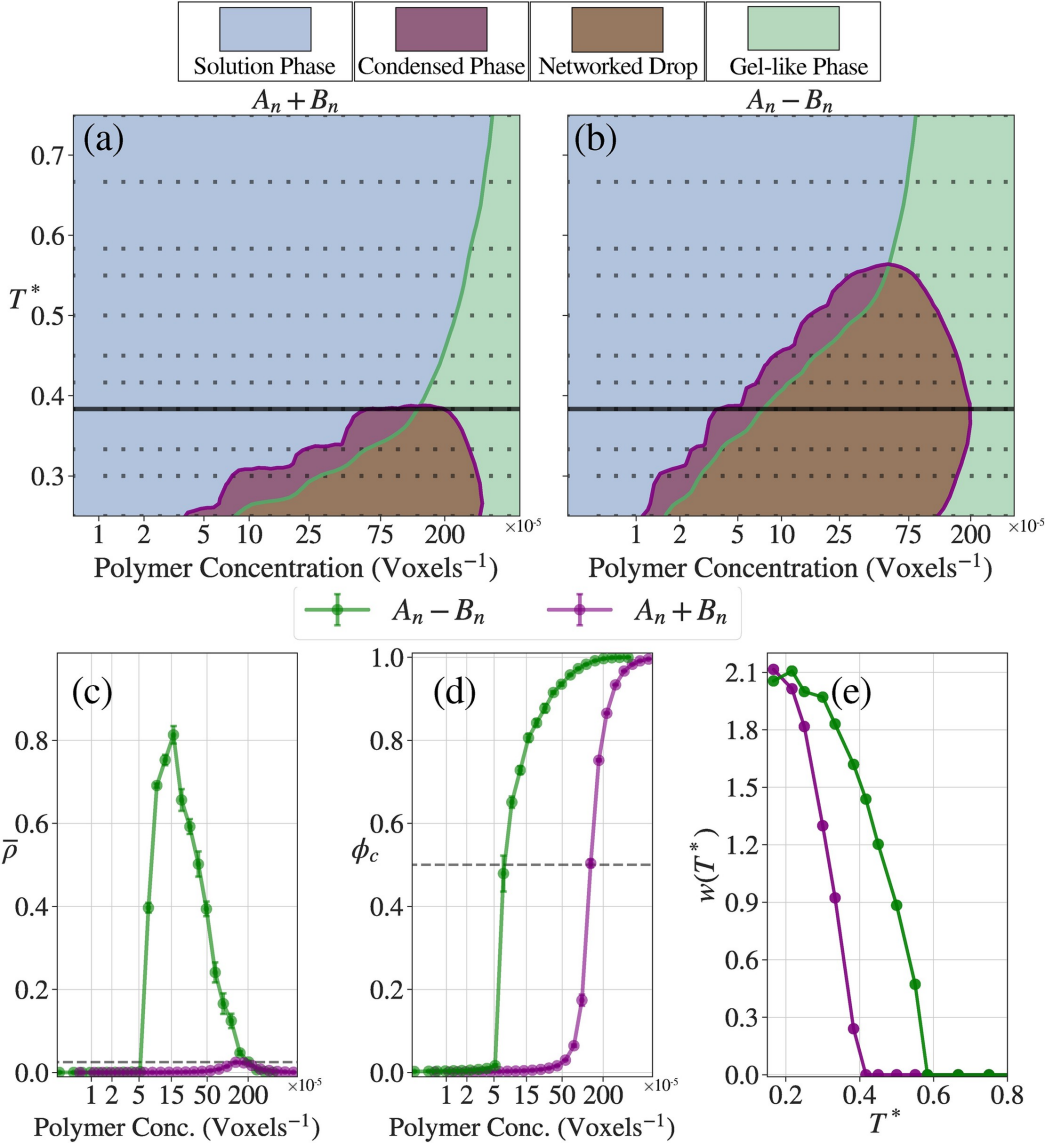
Phase behavior of the linear multivalent systems. (a, b) Phase diagrams for the A_*n*_+B_*n*_ and A_*n*_-B_*n*_ systems, respectively. The purple line is a 2-dimensional linear interpolation for $\bar{\rho }$ = 0.025, and the area encapsulated by the purple line are where the systems have large density inhomogeneities and are thus considered to be phase separated. The green line is a 2-dimensional linear interpolation for ϕ_c_ = 0.5 and thus is the proxy for the percolation line. (c, d) $\bar{\rho }$ and ϕ_c_ curves as a function of concentrations at T* = 0.383 (solid lines in (a) and (b)). (e) Width of the two-phase regime, *w*(T*), as a function of the reduced temperature. Not only does the A_*n*_-B_*n*_ system have a higher critical temperature (T* ~ 0.6 vs. T* ~ 0.4), but also has a wider two-phase regime than the A_*n*_+B_*n*_ system.

**Fig 7** shows phase diagrams for the A_n_+B_n_ and A_n_-B_n_ systems calculated using data from LASSI-based simulations. In panels (a) and (b), the ordinate quantifies the reduced temperature T* calculated as $T^{*}=\frac{k_{B}T}{\varepsilon }$ where ε is the effective energy of pairwise interactions between stickers from the A_n_ and B_n_ blocks. Panel (a) shows results for the A_n_+B_n_ system. The bulk concentration in the A_n_+B_n_ system is quantified along the abscissa as $c_{\mathrm{bulk}}=\sqrt{c_{A_{n}}c_{B_{n}}}$ where $c_{A_{n}}\;\mathrm{and}\;c_{B_{n}}$ are the bulk concentrations of A_n_ and B_n_, respectively. Panel (b) shows the phase diagram for the A_n_-B_n_ system where the abscissa represents the bulk concentration of this system.

Experiments show that the driving forces for phase separation are roughly twice as large for the full-length FUS compared to the system comprising of a 1:1 mixture of PLDs and RBDs [24]. This feature is recapitulated in LASSI simulations. For example, the width of the two-phase regime is larger for the A_n_-B_n_ system compared to the A_n_+B_n_ system for all values of T* as shown in panel (e) of **Fig 7**. The critical temperature is higher for the A_n_-B_n_ vs. A_n_+B_n_ system ($T_{c}^{*}\approx 0.56$ vs. $T_{c}^{*}\approx 0.36$, respectively). The valence of stickers is the main determinant of the concentration at the critical point whereas the interactions mediated by spacers determine the density inhomogeneities and the critical temperature. The impact of longer chains and increased valence of stickers per chain is also evident from the percolation threshold, which is crossed at a bulk protein concentration that is two-fold lower for the A_n_-B_n_ system when compared to the A_n_+B_n_ system, across all the simulation temperatures. Differences between the two systems are also evident in the degree of cooperativity of phase separation and the percolation transition as shown in panels (d) and (e) of **Fig 7**.

For each system, the intersection of the percolation threshold line with the two-phase regime shows that the dense phase predominantly forms a percolated droplet–panels (a) and (b) in **Fig 7**. Therefore, while phase separation without percolation is realizable, this is not the dominant scenario for associative polymers, where phase separation and percolation are typically conjoined to give rise to percolated droplets. The density of proteins in these percolated droplets is governed by the interaction strengths, modulated by T* and the effective solvation volumes of spacers [28, 29]. Unlike homopolymers, which comprise entirely of stickers or spacers depending on the solvent quality, associative polymers encompass a mixture of stickers and spacers. Stickers provide the driving forces for networking via reversible crosslinks and spacers determine whether or not these driving forces lead to phase separation via condensation. Indeed, the importance of sticker-driven percolation is evidenced in the persistence of percolated networks for both systems at high values of T*.

The observation that dense phases form percolated droplets has several implications: (1) on timescales that are concordant with or smaller than the average lifetime of physical crosslinks between stickers, the condensates will have elastic properties; this will be replaced by viscous behavior on timescales that are longer than the average lifetime of physical crosslinks [96]; (2) accordingly, condensates will have an intrinsic tendency for viscoelasticity [97] and long-lived crosslinks will cause hardening behavior as has been observed in many systems [1, 6, 7, 11, 22, 24, 39–41]; (3) the extent of crosslinking above the percolation threshold will change continuously with concentration [16, 29], and this will govern the overall structure, internal dynamics, and porosity of condensates; (4) reactions within condensates are likely to be constrained by the network topology formed as a result of inter-sticker interactions [98]; these constraints can create a variety of interesting kinetic signatures for reactions [99], including temporal memories as has been demonstrated recently for a system that undergoes thermoresponsive phase behavior [66]. Clearly, any description of biochemical reactions within condensates has to account for the structural and dynamical attributes of percolated droplets that are best described as network fluids.

### Move set frequencies and diagnostics of converged simulations

We used results from simulations of linear multivalent protein system to assess the design of LASSI. The frequencies of the different move sets for simulations of the linear multivalent protein system are summarized in **Table 2**. Considerations that go into the design of move sets include the achievement of converged equilibrium distributions, with maximal computational efficiency, for each bulk concentration. Details of these considerations were described in the methods section. Diagnostics from short simulations are often useful to optimize the move set frequencies especially if multiple short trials are performed using very different starting configurations.

**Table 2:** Move frequencies according to their types. They are normalized to the sum of all frequencies used in each simulation.

	FUS-like system	N130 + rpL5 system
Cluster translation move	1	1
Chain translation move	10	10
Rotation move	100	100
Local move	250	250
Reptation move	0	50
Double pivot move	50	10

**Fig 8:**
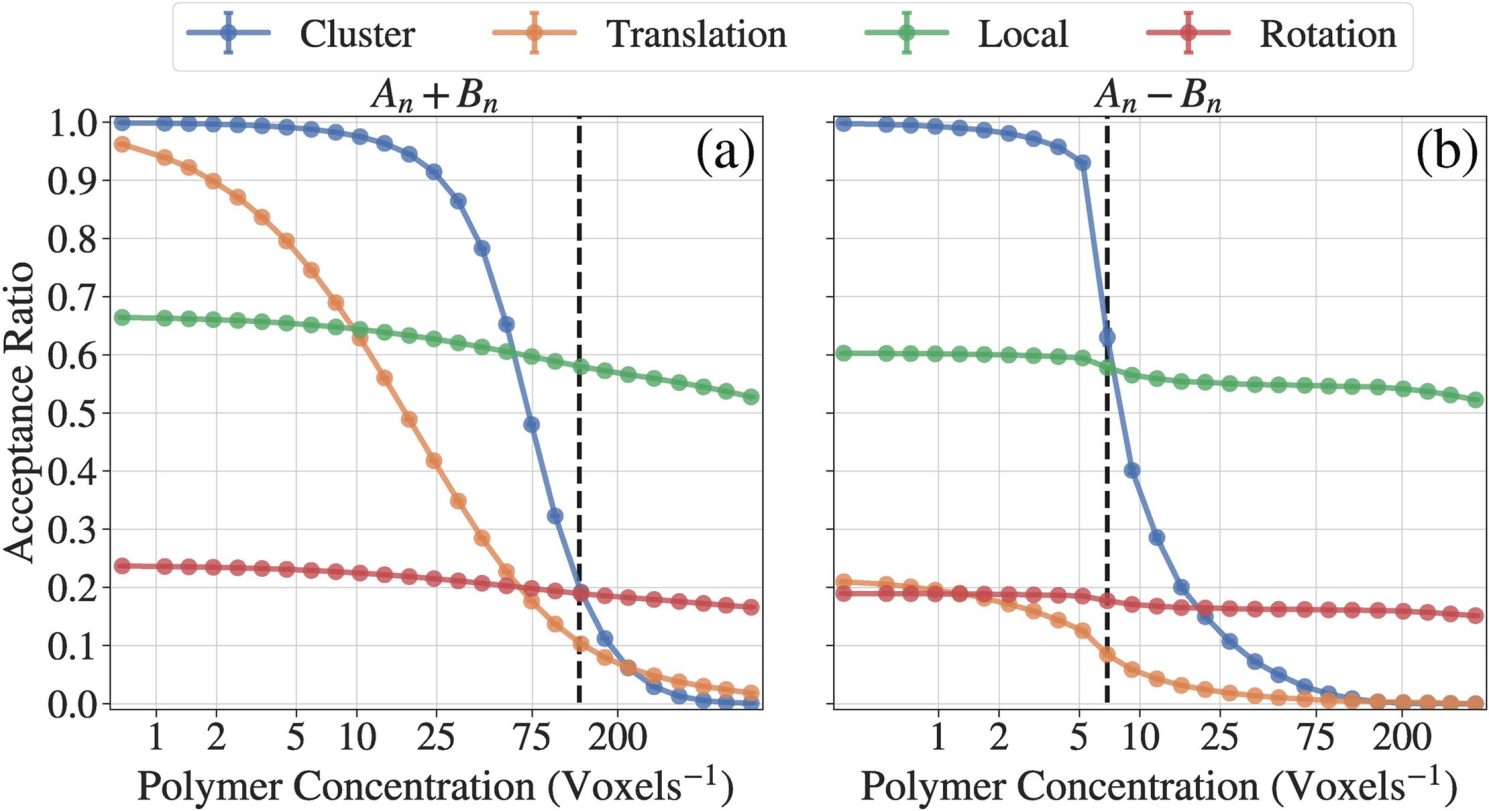
Analysis of acceptance ratios for different move sets. Curves with different colors indicate acceptance ratios of different types of moves. The dashed lines show the saturation concentrations. The data are obtained from simulations with T* = 0.383. (a) Acceptance ratio data for the A_*n*_+B_*n*_ system. (b) Acceptance ratio data for the A_*n*_-B_*n*_ system.

**Fig 8** shows the concentration dependence of acceptance ratios for each of the move set types, diagnosed for simulations of the A_n_+B_n_ and A_n_-B_n_ systems. The acceptance ratios show similar qualitative trends for both systems, even though there are clear quantitative differences. The move with the highest acceptance ratio in the dense regime is the double pivot move, signifying that the systems are transitioning into a pure polymer melt. The second most accepted move is the local move; extrapolating from the higher concentrations it is expected that the acceptance of local moves should also become small and that the double pivot move will be the most dominant way to alter chain configurations, since even the move of an individual monomer will require that multiple monomers from multiple chains are moved simultaneously. Both systems have similar qualitative trends for the translation move where we see a transition from being accepted at low concentrations to not being accepted at higher concentrations. Since the proteins in the A_n_-B_n_ system are twice long as the A_n_+B_n_ system, the absolute acceptance ratio of the translation move is always lower in the A_n_-B_n_ system.

**Fig 9:**
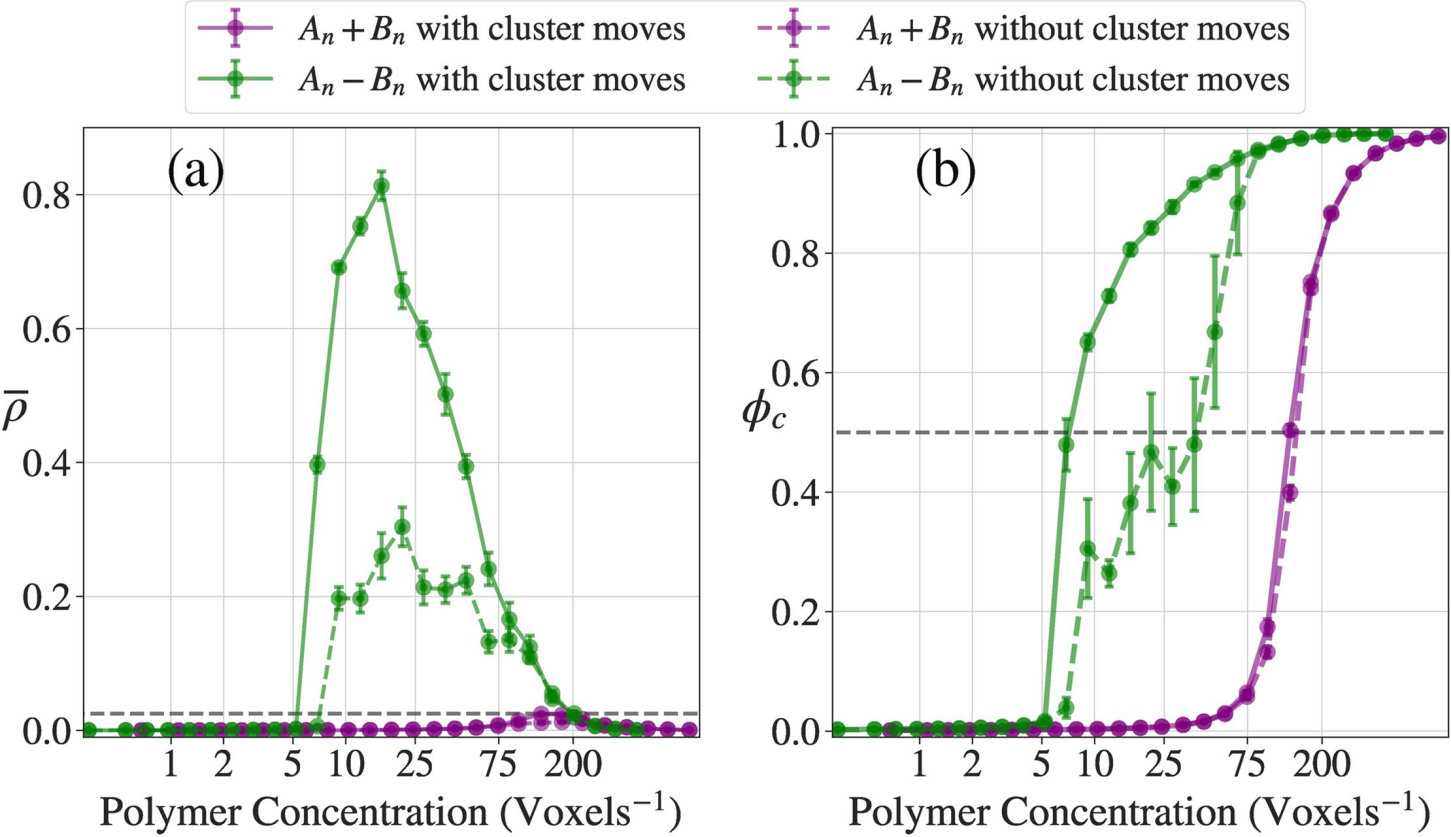
Importance of cluster translation moves. (a) $\bar{\rho }$ and (b) ϕ_c_ curves for the A_*n*_+B_*n*_ (purple) and A_*n*_-B_*n*_ systems (green) at T* = 0.383. The solid lines are identical with the curves in panels (c) and (d) of **Fig 7**. The dotted lines show the simulation results under the same system conditions but the frequency for cluster translation moves is set to zero. Not only do the systems phase separate and percolate at higher saturation concentrations, but also we can see that both percolation and separation are suppressed highly. Furthermore, errors are generally higher, due to the systems being highly dependent on the initial conditions of the system.

Analysis of acceptance ratios of different move sets within droplets will be helpful for estimating correlation lengths and amplitudes of conformational and concentration fluctuations within droplets. Cluster moves have high acceptance ratios in the dilute regime whereas the acceptance ratio nearly vanishes as the concentration increases. This is intuitive since the likelihood of steric clashes increases with a decrease in available volume and this is coupled to the simultaneous increase in the fraction of molecules in the largest cluster. We note here that the cluster moves have the most dramatic change in acceptance ratios from values near 1 to values near 0. However, the apparent inefficiency of cluster moves in dense configurations cannot be used as a rationale to quench such moves. In fact, as shown in panel (a) of **Fig 9**, phase separation, diagnosed in terms of $\bar{\rho }$, is suppressed if cluster moves are not part of the move set. This highlights the importance of cluster moves for generating *bona fide* phase separation as these facilitate coalescence that leads to condensation.

### Mixtures of N130 and the rpL5 peptide as an example of a branched multivalent protein system

LASSI can also be deployed to study the phase behavior of branched multivalent proteins that undergo phase separation via obligate heterotypic interactions. Examples of branched multivalent proteins are molecules that form symmetric, stable oligomers such as nucleophosmin 1 (NPM1) and synthetic systems such as the corelets designed by Bracha *et al*. [100]. NPM1 is a key molecule within the granular component (GC) of the nucleolus [101]. Three coexisting layers define the nucleolus and the GC is the outermost layer. Within the GC, NPM1 appears to form facsimiles of percolated droplets in complex ribosomal proteins with Arg-rich motifs [17, 30]. A minimalist system that mimics the phase behavior of the GC comprises of a truncated version of NPM1, referred to as N130, and an Arg-rich peptide, designated as rpL5 [31–33]. Both NPM1 and N130 form symmetric pentamers in the presence of Arg-rich peptides [102]. The pentamer formed by the association of folded domains serves as a scaffold for displaying disordered C-terminal tails that are defined by two distinct acidic tracts. The system also features an N-terminal disordered region with a well-defined acidic motif.

The FUS system is an example of a protein that undergoes phase separation via obligate homotypic interactions. This implies that the interactions necessary and sufficient for driving phase separation are encoded within the sequence of FUS and the strengths of these interactions can be modulated by changes to solution conditions. The N130 + rpL5 system is an example of a system that undergoes phase separation mainly via obligate heterotypic interactions that involve interactions between residues in the acidic tracts of N130 and the Arg motifs of rpL5. This could be viewed as an example of phase separation via complex coacervation, providing the heterotypic interactions are purely electrostatic in nature [31–33]. However, in general, there is likely to be combination of long- and short-range interactions that contribute to the spectrum of heterotypic interactions, and hence we refer to this class of molecules as drivers of phase separation via obligate heterotypic interactions.

In addition to demonstrating the applicability of the LASSI engine for simulations of branched systems, we use the analysis as an opportunity to highlight key conceptual features of multicomponent systems that undergo phase separation via obligate heterotypic interactions. There are three main features that are borne out in the analysis: (1) For fixed temperature, the order parameters are the concentrations of the proteins that drive phase separation via obligate heterotypic interactions. In such systems, the coexistence curves, *i*.*e*., the binodals, will have a closed loop form. These will be ellipses for two components and *n-*ellipsoids for systems that involve up to *n* obligate heterotypic interactions to drive phase separation. (2) The systems will support reentrant phase behavior as has been reported recently for protein-RNA mixtures that undergo phase separation via obligate heterotypic interactions [103]. (3) The apparent saturation concentration of a component molecule in a system that undergoes phase separation via obligate heterotypic interactions will show non-trivial dependencies on its bulk concentration. These dependencies are governed directly by the slopes of the tie lines that pass through the point corresponding to the bulk concentration and intersect the binodal at coexisting concentrations that equalize the chemical potentials of all species in the dense and dilute phases. Here, we use the example of the N130 + rpL5 system to showcase the three central features of phase diagrams for systems that undergo phase separation via obligate heterotypic interactions.

**Fig 10:**
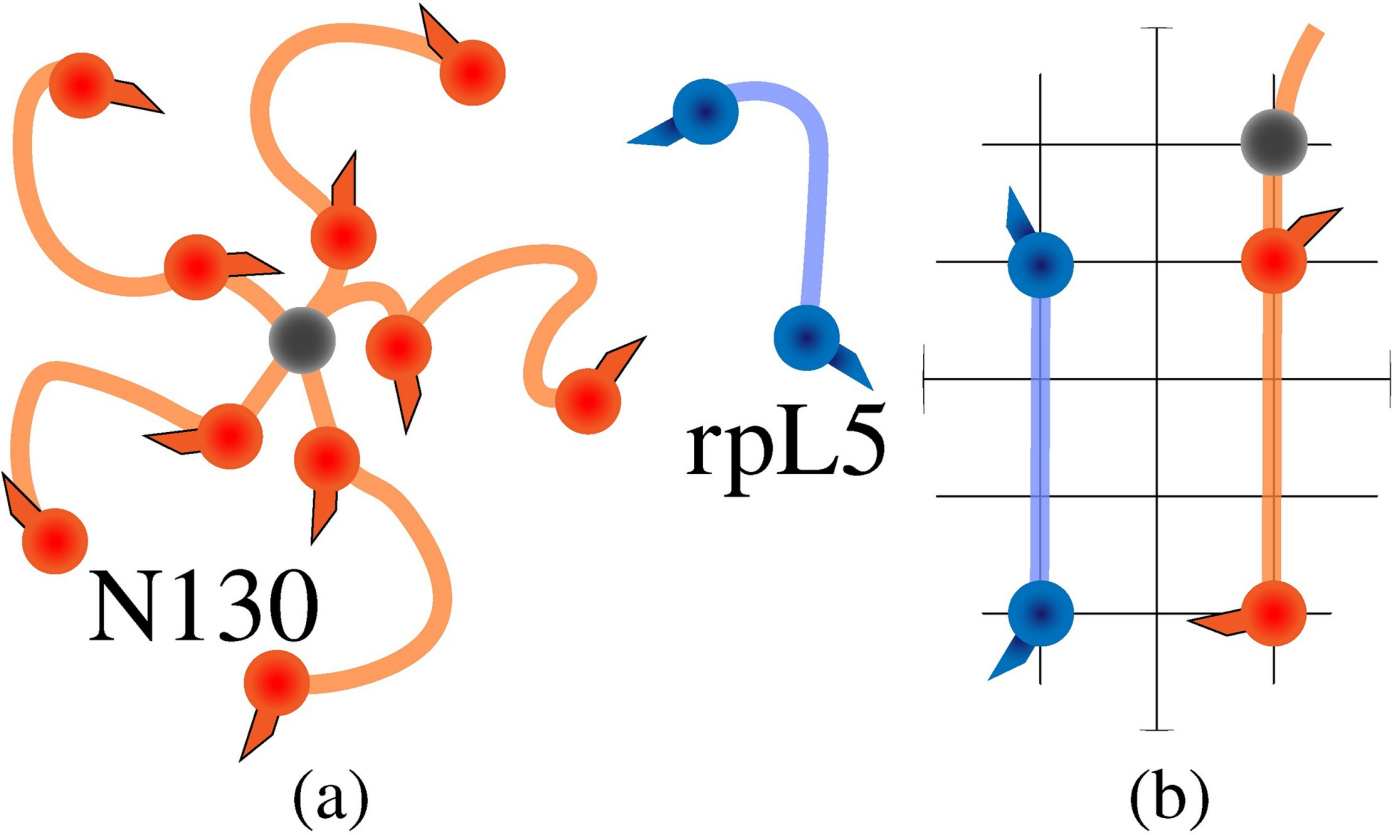
Architecture of an archetypal branched multivalent system. (a) Schematic to depict the overall architecture. The pentamer with 10 orange stickers represents the N130 molecule where the gray central oligomerization domain is modeled as a neutral spacer monomer, and the rpL5 peptide is modeled as a linear molecule with 2 blue stickers. (b) Relevant length scales for the architecture (see also **Table 1**). For the rpL5 molecule a linker length of 3 was chosen between the two stickers, and for the N130 molecule the first sticker (modeling the A1 tract) is 1 lattice site away from the hub spacer whereas the second sticker (modeling the A2 tract) is 3 lattice sites away from the first sticker.

In the LASSI representation, N130 pentamers with disordered tails are modeled using a five-armed structure. This approach follows the strategy of Feric *et al*. [30], which was based on the fact that pentamers do not dissociate under conditions where NPM1 / N130 undergo phase separation. Each arm comprises of two sticker sites to mimic the presence of the A1 and A2 acidic tracts within the disordered tails of NPM1 / N130. Therefore, each N130 pentamer displays a total of ten sticker sites. The spacers between each A1 tract and the N130 core as well as between each pair of A1 and A2 tracts on a disordered tail are phantom tethers, which means that their effective solvation volumes [29] are set to zero. Each rpL5 peptide has two sticker sites corresponding to the two Arg-rich motifs along the sequence. Schematic representations of the coarse-grained architecture used for N130 and rpL5 are shown in **Fig 10**, and the move set frequencies are summarized in **Table 2**.

### Percolation lines have parabolic shapes

**Fig 11:**
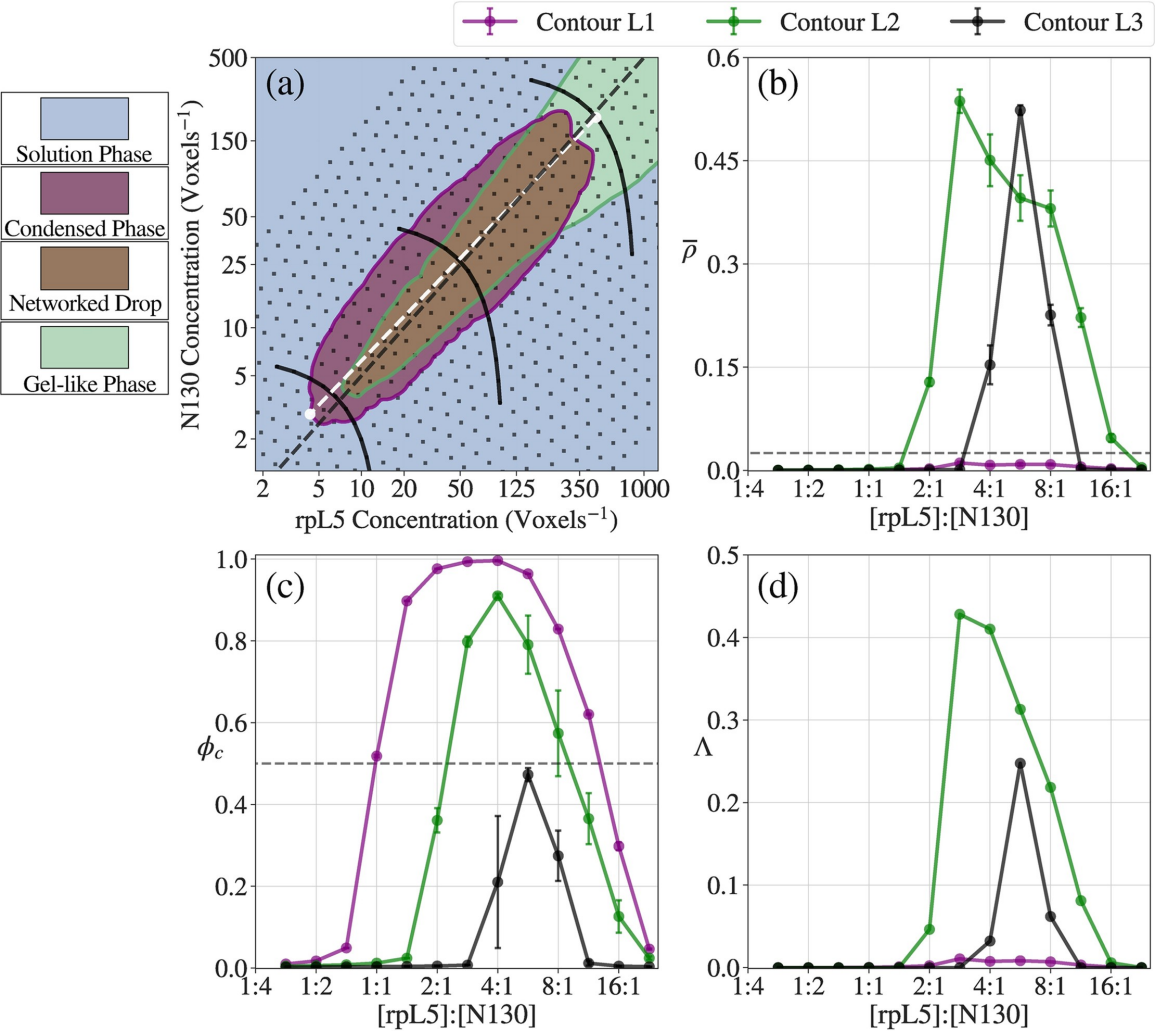
Phase behaviors of the branched multivalent systems for T* = 0.25. (a) Full phase diagram, where the purple line denotes the proxy for the binodal and the green line is the proxy for the percolation line (see also the caption for **Fig 7**). The phase-separated region has an elliptical shape and we have a closed loop, which demonstrates re-entrant phase behavior, whereas the percolation line has a conical shape extending into much higher densities. The solid black lines denote contours of constant total concentration where L1 is the lowest concentration and L3 is the highest concentration. Note that both axes are represented in the log scale. (b, c) $\bar{\rho }$ and ϕ_c_ curves as a function of relative stoichiometric ratio of N130 and rpL5 along the constant-concentration contours. (d) Plot of Λ vs. the apparent stoichiometry along lines L1, L2, and L3.

The percolation line, constructed as a function of the concentrations of two multivalent molecules, has an overall parabolic shape (panel (a) of **Fig 11**). This feature may be explained as follows: Let *f*_1_ and *f*_2_ denote the fractions of N130 and rpL5 molecules that are bound in a network; *s*_1_ and *s*_2_ will denote the valence of stickers on N130 and rpL5, respectively; for the current implementation of the N130 + rpL5 system, *s*_1_ = 10 and *s*_2_ = 2. Based on the Flory-Stockmayer theory [49, 51], the percolation threshold is crossed when *f*_1_*f*_2_(*s*_1_−1)(*s*_2_−1) > 1. If we keep (*s*_1_−1)(*s*_2_−1) constant, the threshold concentration for percolation will be governed by the product *f*_1_*f*_2_. Accordingly, if there is a large excess of N130 (component 1) compared to rpL5 (component 2), then *f*_1_ → 0 and *f*_2_ → 1, and the system does not undergo a percolation transition. In this scenario, every rpL5 molecule is crosslinked to two sticker sites from one or two N130 molecules. However, since the relative stoichiometry favors N130 molecules, there is a vast excess of un-crosslinked N130 molecules and the network cannot grow. Percolation is also inhibited when the converse situation arises, *i*.*e*., when there is a large excess of component 2 with respect to component 1. Accordingly, the percolation line takes on a parabolic form in the plane defined by the concentrations of N130 and rpL5.

### Binodals for systems defined by obligate heterotypic interactions will form closed loop ellipses

Given that the phase behavior of the N130 + rpL5 system is driven by heterotypic interactions involving the A1 / A2 tracts from the N130 tails and the Arg-motifs from rpL5, we constructed binodals by keeping the simulation temperature fixed and varied the concentrations of N130 and rpL5 molecules. Phase diagrams defined by N130 concentration along the abscissa and rpL5 concentration along the ordinate are shown in panel (a) of **Fig 11**. The general shape of the binodal is comparable with that of the experimentally determined phase diagram [33], even though direct comparison is not straightforward because the scarcity of experimental data points does not yield a full binodal.

The phase boundary, defined by the density transition, is an ellipse that forms a closed loop in the plane defined by the concentrations *c*_1_ and *c*_2_ of N130 and rpL5, respectively. In associative polymers, the phase behavior is governed by the affinity between stickers, the valence of stickers, and the effective solvation volumes of spacers [28, 29]. For fixed *c*_1_ that intersects the two-phase regime an increase in *c*_2_ will lead to an entry into the two-phase regime caused by a density transition as *c*_2_ approaches *c*_1_. However, as *c*_2_ increases well beyond *c*_1_, the joint system exits the two-phase regime. This is because phase separation is driven by obligate heterotypic interactions and while there is a growing excess of rpL5 molecules there are not enough N130 molecules to drive the density transition via inter-sticker interactions. Similar reasoning applies to describe the reentrant behavior that will result by keeping *c*_2_ fixed at a value that intersects the two-phase regime and increasing *c*_1_.

Taken together, the parabolic percolation lines and elliptic forms for two-phase regimes define conic sections that highlight reentrant phase behavior whereby fixing the concentration of component 1 and increasing the concentration of the second species can lead to phase separation and percolation at a low threshold concentration of component 2 and exit into the one-phase, non-percolated regime beyond a second higher threshold concentration for component 2. This type of reentrant phase behavior, will be a general feature of multicomponent systems that undergo phase separation via obligate heterotypic interactions; indeed, reentrant phase behavior has been reported for a model protein + RNA system [103].

### Apparent stoichiometric ratios can be different from effective stoichiometric ratios

Stoichiometry of molecules that drive phase separation is another key parameter that determines the functions of biomolecular condensates formed by multicomponent systems [104]. The apparent stoichiometric ratio is calculated as the ratio of the concentrations of stickers of types *s*_1_ and *s*_2_ for N130 and rpL5, respectively such that $\nu_{12}^{\mathrm{app}}=\frac{c_{s1}}{c_{s2}}$. However, the effective stoichiometric ratio $\nu_{12}^{\mathrm{eff}}$ can be different from $\nu_{12}^{\mathrm{app}}$ if excluded volume effects modulate the effective concentration of stickers. We fit an ellipse to the two-phase boundary and determined the major axis of this ellipse. The effective stoichiometric ratio should be unity along the major axis. As shown in panel (a) of **Fig 11**, the major axis deviates from the line along which $\nu_{12}^{\mathrm{app}}=1$. Therefore, $\nu_{12}^{\mathrm{app}}\neq \nu_{12}^{\mathrm{eff}}$ and angle between the major axis and the line along which $\nu_{12}^{\mathrm{app}}=1$ quantifies the impact of excluded volume on changes to effective concentrations of stickers that in turn modifies the stoichiometric ratios.

The synergy between stoichiometry and phase behavior can be analyzed by quantifying the order parameter $\bar{\rho }$ and the topological parameter ϕ_c_ as a function of apparent stoichiometry for fixed bulk concentration. Along each gray line in panel (a) of **Fig 11** the total concentration defined as *c*_bulk_ = (*c*_1_*c*_2_)^½^ is fixed, although the stoichiometries will vary. The mean values of *c*_bulk_ along L1, L2, and L3 are 2.09×10^−2^ (voxel^–1^), 2.46×10^−3^ (voxel^–1^), and 3.33×10^−4^ (voxel^–1^), respectively and the value of $\nu_{12}^{\mathrm{app}}$ ranges from 0.36 to 22.62 along each of L1, L2, and L3. Panels (b) and (c) in **Fig 11** show the variation of $\bar{\rho }$ and ϕ_c_ as $\nu_{12}^{\mathrm{app}}$ increases along L1, L2, and L3, respectively. Along L1, the value of $\bar{\rho }$ is essentially zero irrespective of stoichiometry because L1 lies is outside the two-phase regime. However, a system spanning percolated network forms for stoichiometries in the range 1.2 ≤ ν_12_ ≤ 13 along L1. This is because the concentrations of both components are well above the percolation threshold along L1 thus ensuring that stickers readily find one another even without a density transition. In direct contrast, along L3, we observe phase separation, characterized by values of $\bar{\rho }$ > 0.025 for a range of stoichiometries, but none of these support the formation of a percolated droplet (ϕ_c_ < 0.5 for all stoichiometries). Along L2, we observe phase separation for stoichiometries in the range 1.15 ≤ ν_12_ ≤ 16 and percolation for stoichiometries in the range 2.14 ≤ ν_12_ ≤ 11.3 such that phase separation enables the formation of a percolated droplet.

In panel (d) of **Fig 11**, we introduce a new structural parameter Λ, which we define as a convolution of $\bar{\rho }$ and ϕ_c_ such that $\Lambda =\bar{\rho }⊗\phi_{c}$. Here, the convolution is calculated as a logical AND gate, which becomes a simple product. The parameter Λ quantifies the convolution of the density and network transition and provides an estimate of the extent to which the phase separation and percolation are coupled as the apparent stoichiometry is varied for a fixed bulk concentration. The profile of Λ is reminiscent of profiles measured by Case *et al*. [104] for the dwell time of signaling molecules as a function of stoichiometric ratios that govern the formation of condensates at membranes. This suggests that dwell times, which are experimentally accessible parameters, might actually be proxies for the structural features of the condensates as measured by the convolution between phase separation and percolation and the extent of network formation within the condensate.

The key finding is that the combination of the bulk concentration and stoichiometric ratio (as opposed to stoichiometry alone) will determine the quench depth into the two-phase regime. This in turn determines whether a system-spanning network forms without phase separation or if phase separation enables the formation of a droplet-spanning network. The structure of condensates and the overall phase behavior cannot be fully described in terms of *c*_bulk_ or ν_12_ alone. Instead, this requires the consideration of both parameters jointly and relative to the quench depth, which refers to the location in the two-phase regime and with respect to the percolation line. This is important because the extent of crosslinking and the time scales associated with crosslinks will determine the material properties of the condensate. This in turn should contribute to parameters such as the dwell times of clients within condensates [104].

### Saturation concentrations need not be fixed parameters in multicomponent systems

The concept of a saturation concentration is one of the defining hallmarks of phase separation [2, 27]. For fixed solution conditions, phase separation in a closed two-component system (or pseudo one-component system) comprising of a protein and solvent is realized when the bulk concentration of the protein denoted as *c* exceeds a saturation concentration denoted as *c*_sat_. The presence of a saturation concentration can be quantified by measuring the concentration of protein in the coexisting dilute phase as *c* increases. This value will not go above *c*_sat_. Strikingly, the presence of a saturation concentration has been confirmed in living cells for model disordered proteins by Brangwynne and coworkers using optogenetic tools in mammalian cells [100, 105] and by Khan *et al*. [106] using yeast as a model system.

**Fig 12:**
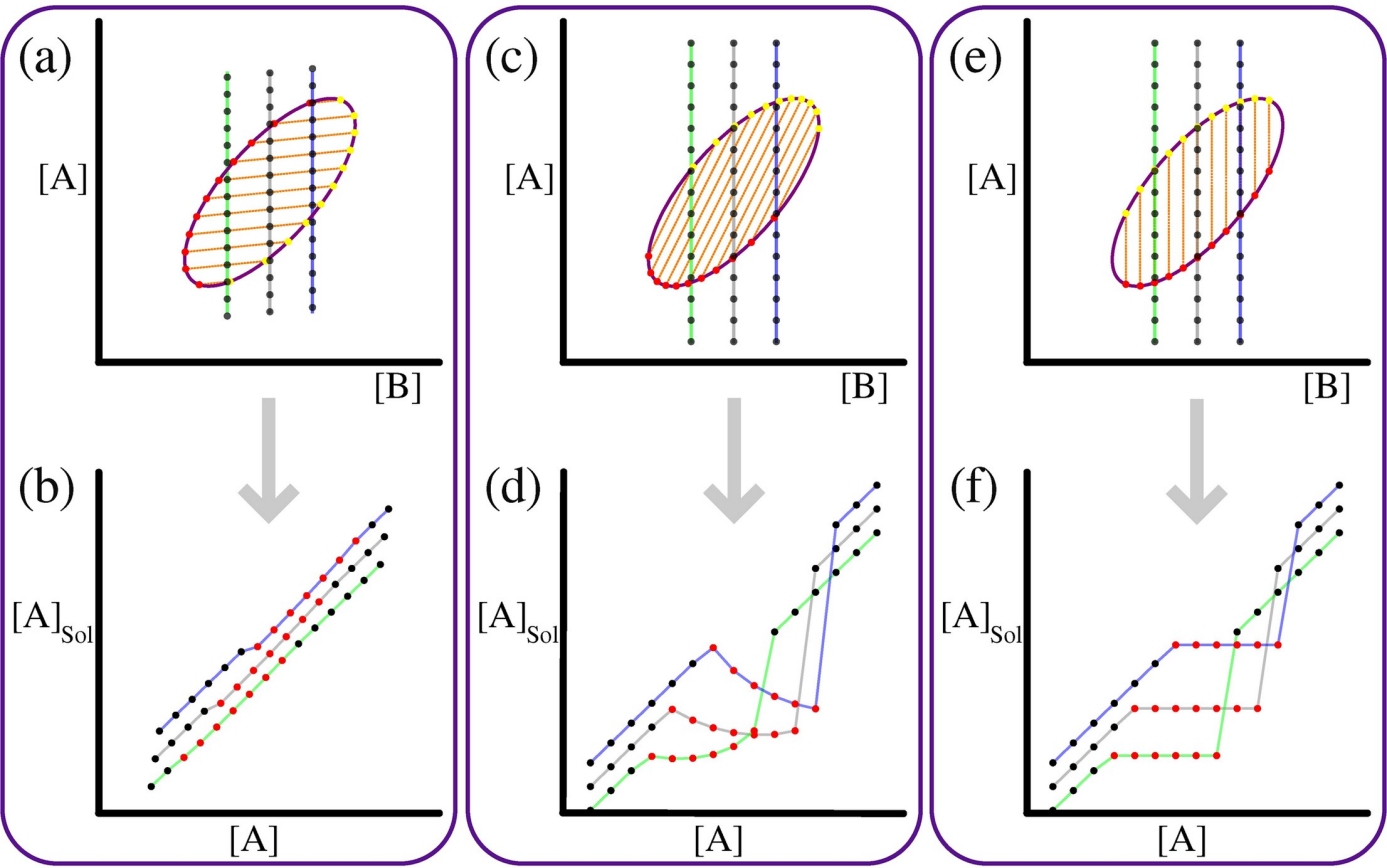
Slopes of tie lines within elliptic binodals are important for systems that undergo phase separation via *obligate heterotypic interactions*. The ellipse is drawn to fit the locus of points based on the value $\bar{\rho }$ that meets our criteria for a density transition (see main text). Data for constructing the ellipse were taken from simulations of the N130 + rpL5 system–see **Fig 11(A)**. This ellipse is used to assess the impact of slopes of tie lines for a two-component system comprising of macromolecule A that undergoes phase separation via obligate heterotypic interactions with macromolecule B. (a) Ellipse with nearly horizontal tie lines. The vertical lines shown in green, grey, and blue correspond to fixed values for [B] along the abscissa. As [A] increases, the system traverses across the two-phase regime, delineated by the ellipse, starting outside the ellipse, crossing the ellipse, and exiting the ellipse at high concentrations of A. (b) For each fixed value of [A], the plot shows how [A]_Sol_ varies with [A]. The red points on each curve were extracted from within the two-phase regime, whereas the black points lie outside the two-phase regime. Clearly, [A]_Sol_ does not stay fixed as [A] increases. (c) Equivalent plot to that shown in panel (a) for the tie lines that we obtain for the N130 + rpL5 system. (d) Equivalent plot to that shown in panel (b). Note the non-linear variation of [A]_Sol_ as [A] increases. (e) Ellipse annotated with vertical tie lines. In this case, phase separation of [A] does not depend on obligate heterotypic interactions with B, but B can bind to A and has a choice of binding preferentially to A in either the dense or dilute phase. Here, A becomes the macromolecule and B the ligand. (f) Preferential binding of the ligand to the macromolecule in its dilute phase will shift the saturation concentration, assessed in terms of [A]_Sol_, upward and this shift will depend on [B]. Accordingly, the plateau value of [A]_Sol_ in the two-phase regime shifts to higher values for higher values of [B].

If the protein whose phase behavior is being interrogated is part of a system where *obligate heterotypic interactions* drive phase separation, then whether or not the concept of a saturation concentration continues to be valid will depend on the nature of the binodals. We illustrate this by coopting the elliptic phase boundary from **Fig 11** for a three-component system that comprises of N130, (component A), rpL5 (component B), plus a solvent that is implicit in the LASSI simulations. This 3-component system may be thought of as a pseudo two-component system. For fixed temperature, the top row of panels (a)-(c) in **Fig 12** show three types of elliptical, closed loop binodals. These are constructed in a plane where [B] increases along the positive direction of the abscissa and [A] increases along the positive direction of the ordinate. The bottom row in each panel shows the result to be expected were we to measure the concentration of A in the dilute phase, designated as [A]_Sol_, as the bulk concentration [A] is varied. In each of these measurements, the concentration of B is fixed at a specific value.

### Slopes of tie lines within the elliptic binodals determine how [A]_Sol_ varies with [A] in the two-phase regime

For concentrations of A and B that place the pseudo two-component system in the two-phase regime–red points along each of the curves in the bottom rows of panels (a)–(c)–we find that [A]_Sol_ can change as [A] increases. If the tie lines are horizontal or nearly horizontal, then [A]_Sol_ will vary linearly with [A]. Non-linear variations of [A]_Sol_ with [A] will result for tie lines with positive or negative slopes. This is shown in panel (b) for tie lines with positive slopes. If the tie lines are essentially vertical, then the standard expectation regarding the invariance of [A]_Sol_ with [A] within the two-phase regime is recovered. However, even in this scenario, the plateau value of [A]_Sol_ will shift upward or downward as the value of [B] increases–the upward shift is shown in panel (c) of **Fig 12**. Here, B acts as a *bona fide* ligand for A, which is the macromolecule. Preferential binding of B to the dilute phase leads to an increase in [A]_Sol_ as depicted in panel (c) of **Fig 12**. Ligand-mediated shifts in saturation concentrations arise due to polyphasic linkage, a phenomenon first introduced by Wyman and Gill [107].

*The main conclusion is that the concept of a saturation concentration*, *as defined for a pseudo one-component system*, *does not transfer over to multicomponent systems where phase transitions are driven by obligate heterotypic interactions*. Instead, the slopes of tie lines or the geometries of tie planes in higher dimensional ellipsoids will have a direct bearing on inferences from measurements where the bulk concentration of a protein or RNA component is varied when condensates are observed and the concentration of the molecule of interest is quantified in the coexisting dilute phase. This insight emerges from our ability to deploy LASSI to compute full binodals for multicomponent systems.

## Discussion

In this work, we have built on the connection between multivalent proteins and associative polymers [44, 45, 98] with their stickers-and-spacers architecture [15, 17, 24, 28, 29, 40, 43, 47] to develop and deploy LASSI, a lattice-based open source computational engine that enables the simulation of system-specific phase diagrams of single and multi-component systems. The foundations of LASSI derive from the formalism of the bond fluctuation model [83, 84, 108]. We demonstrate how canonical ensemble Monte Carlo simulations with appropriately designed move sets and analysis of order parameters derived from the distribution functions allow us to calculate coexistence curves and percolation lines as a function of protein concentration and interaction strengths.

The choice of a lattice-based approach for coarse-graining and modeling phase behavior of multivalent proteins is guided by the advantages of lattice models [109] for polymeric systems. To titrate across the full range of volume fractions, one needs to balance considerations of finite size effects–which requires large numbers of molecules–with large simulation volumes–which makes it difficult to observe density fluctuations that can grow into density inhomogeneities. On lattices the conformational space is discretized and the calculation of interaction potentials can be made to be very efficient through the use of look up tables. Importantly, we have generalized lattice-based simulations to incorporate anisotropic interactions.

LASSI allows us to query the impacts of overall and intrinsic valence of stickers, interaction strengths between stickers, the spatial ranges of these interactions, the effective solvation volumes and lengths of spacers, and protein concentrations. These titrations generate multidimensional phase diagrams. The approaches underlying LASSI have been applied to model a variety of multicomponent systems, including mimics of RNA molecules [24, 28–30, 43, 79]. What is required is the development of approaches that enable systematic coarse-graining and adaptation of machine learning based methods to parameterize interaction potentials [78]. Engineering LASSI to be interoperable to cell-based modeling suites [110] will also allow for larger scale deployment of the overall framework. The calculation of pair and higher order distribution functions should afford multiscale descriptions of the structural organization of molecular components within condensates. The acceptance ratios associated with different move sets and the length scales spanned by distinct move sets open the door to analyzing the dynamics of phase separation, percolation, and the interplay between the two. Another major direction for future application of LASSI is to uncover the determinants of compositional specificity of condensates [1, 12].

## Supporting information

S1 Fig Two-dimensional representation of rotation move. For a given randomly selected monomer (middle orange bead), 33−1 nearest lattice sites (yellow box) are checked for possible interaction candidates, where eligible candidates have a non-zero interaction energy with the selected monomer. In this figure, orange stickers interact with blue stickers and thus this sticker has 3 possible candidates. The end orientational state of the monomer is then picked using the metropolis criterion, which also includes the non-interacting state.
(TIF)

S2 Fig Two-dimensional representation of local move. For a given randomly selected monomer, a new location is proposed by sampling ±2 lattice sites in each coordinate (brown box) and picking a lattice site that is empty. If an empty lattice site is found within a pre-determined number of trials, the numbers of interacting candidates are calculated at the old and proposed location (yellow boxes). Then the move is accepted or rejected using the modified Metropolis criterion that considers orientational bias (see text).
(TIF)

S3 Fig Two-dimensional representation of reptation move. For a given randomly selected chain that has the same linker lengths between each monomer, an end is randomly picked. Then, a version of the local move is performed where the selected end is moved to a new random location that is an empty lattice site within 2 lattice sites in each coordinate (brown box). If an empty site is found within a predetermined number of trials, the number of orientational candidates is calculated for the whole chain in the old and the new configuration (yellow boxes). The modified metropolis criterion is then used to determine if the move is accepted or rejected. Note that since the whole chain is orientationally biased, monomers may have a different orientational state after the move is accepted, as shown in the figure.
(TIF)

S4 Fig Two-dimensional representation of double pivot move. For a randomly selected monomer, a 2×2×2 cube around the monomer is searched for appropriate bridging candidates (brown box), where an appropriate bridging candidate is the next monomer from a different chain, is within a linker length of the selected monomer as shown by the dashed line connecting *i*_*m*_ and (*i*+1)_*n*_. Furthermore, the distance between (*i*+1)_*m*_ and *i*_*n*_ must also be within a linker length as depicted by the upper dashed line. A list of all possible candidates is calculated and then a randomly chosen candidate is used to break and remake covalent bonds. This results in a large conformational change for both polymers. If the selected polymer is not linear, the move is rejected outright.
(TIF)

S5 FigAssessments of finite size effects analyzed in terms of $\begin{array}{c} \tilde{g}(r^{*}) \end{array}$.The pair distributions from **Figs 9(B) and 11** are used to compute the relevant radial distribution functions. This analysis is relevant because the radial distribution functions are used to extract the value of the order parameter that detects the onset of phase separation. For systems where the number of molecules A_n_ + B_n_ molecules is greater than 200, the radial distribution functions $\begin{array}{c} \tilde{g}(r^{*}) \end{array}$ start to deviate from one another only at the lowest temperatures where broken ergodicity becomes an issue. Therefore, for the A_n_ + B_n_ system studied in this calibration, it appears that the numbers of A_n_ + B_n_ molecules have to be greater than 200 in order to obtain reliable information about the phase behavior. (a) $\begin{array}{c} \tilde{g}(r^{*}) \end{array}$ extracted for different numbers of A_n_ and B_n_ molecules for T* = 0.167. (b) $\begin{array}{c} \tilde{g}(r^{*}) \end{array}$ extracted for different numbers of A_n_ and B_n_ molecules for T* = 0.217. (c) $\begin{array}{c} \tilde{g}(r^{*}) \end{array}$ extracted for different numbers of A_n_ and B_n_ molecules for T* = 0.267.(TIF)
