# Supplementary figures and images for: LASSI: A lattice model for simulating phase transitions of multivalent proteins
Source: PLoS Comput Biol. 2019 Oct 21;15(10):e1007028. doi: 10.1371/journal.pcbi.1007028 (PMC6822780; doi:10.1371/journal.pcbi.1007028)

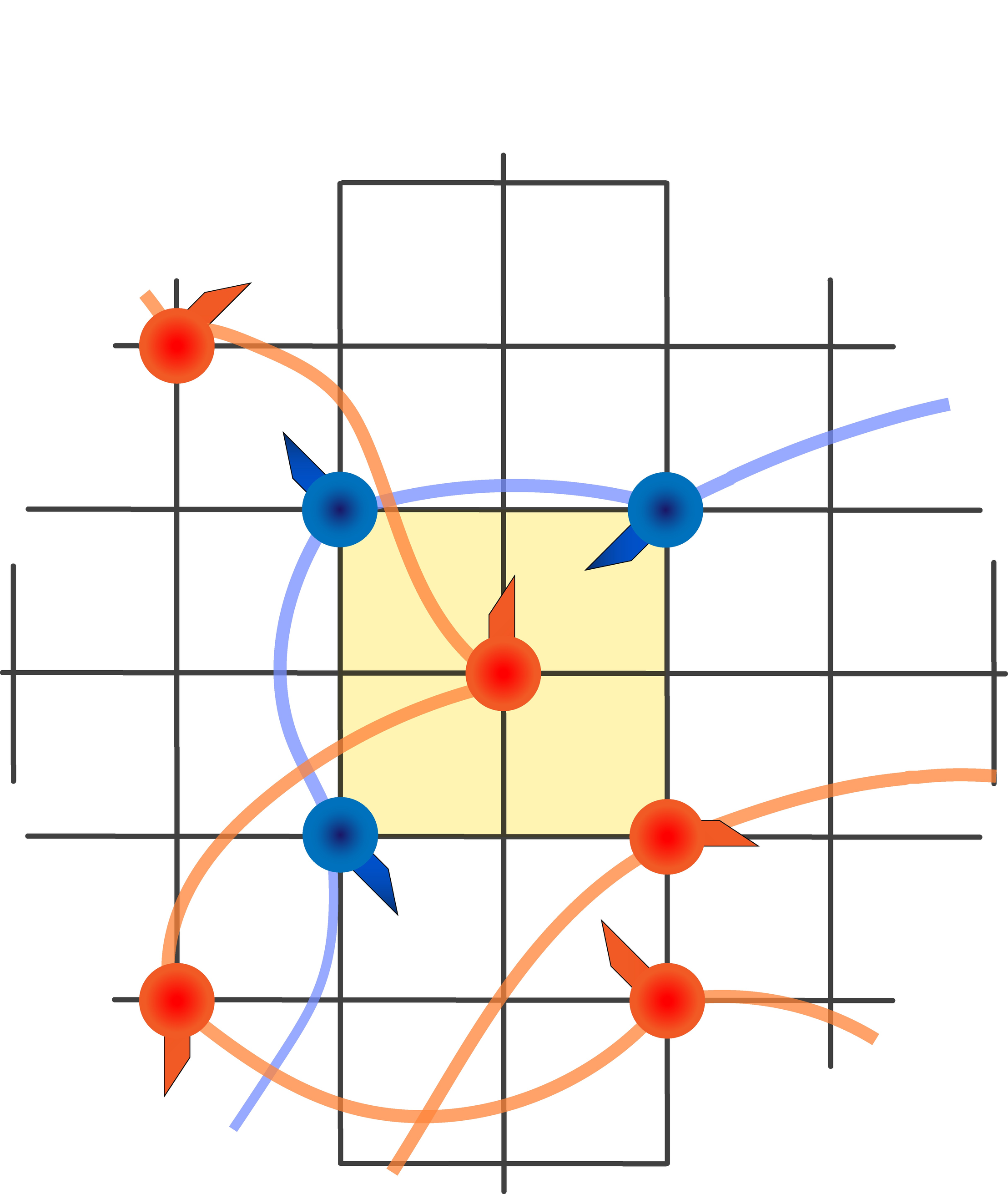

Supplement: S1 Fig — For a given randomly selected monomer (middle orange bead), 33−1 nearest lattice sites (yellow box) are checked for possible interaction candidates, where eligible candidates have a non-zero interaction energy with the selected monomer. In this figure, orange stickers interact with blue stickers and thus this sticker has 3 possible candidates. The end orientational state of the monomer is then picked using the metropolis criterion, which also includes the non-interacting state. (TIF) [file pcbi.1007028.s001.tif]

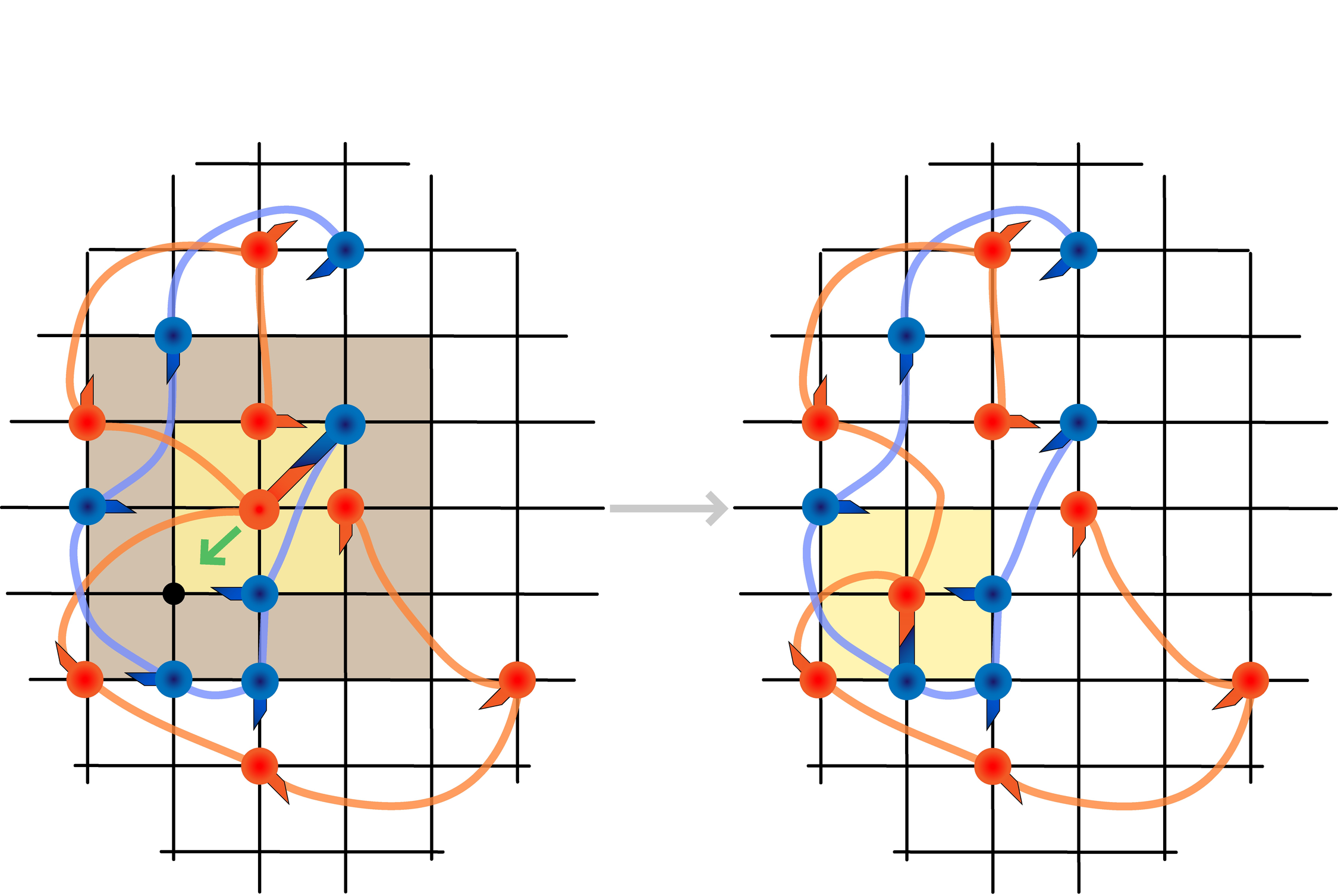

Supplement: S2 Fig — For a given randomly selected monomer, a new location is proposed by sampling ±2 lattice sites in each coordinate (brown box) and picking a lattice site that is empty. If an empty lattice site is found within a pre-determined number of trials, the numbers of interacting candidates are calculated at the old and proposed location (yellow boxes). Then the move is accepted or rejected using the modified Metropolis criterion that considers orientational bias (see text). (TIF) [file pcbi.1007028.s002.tif]

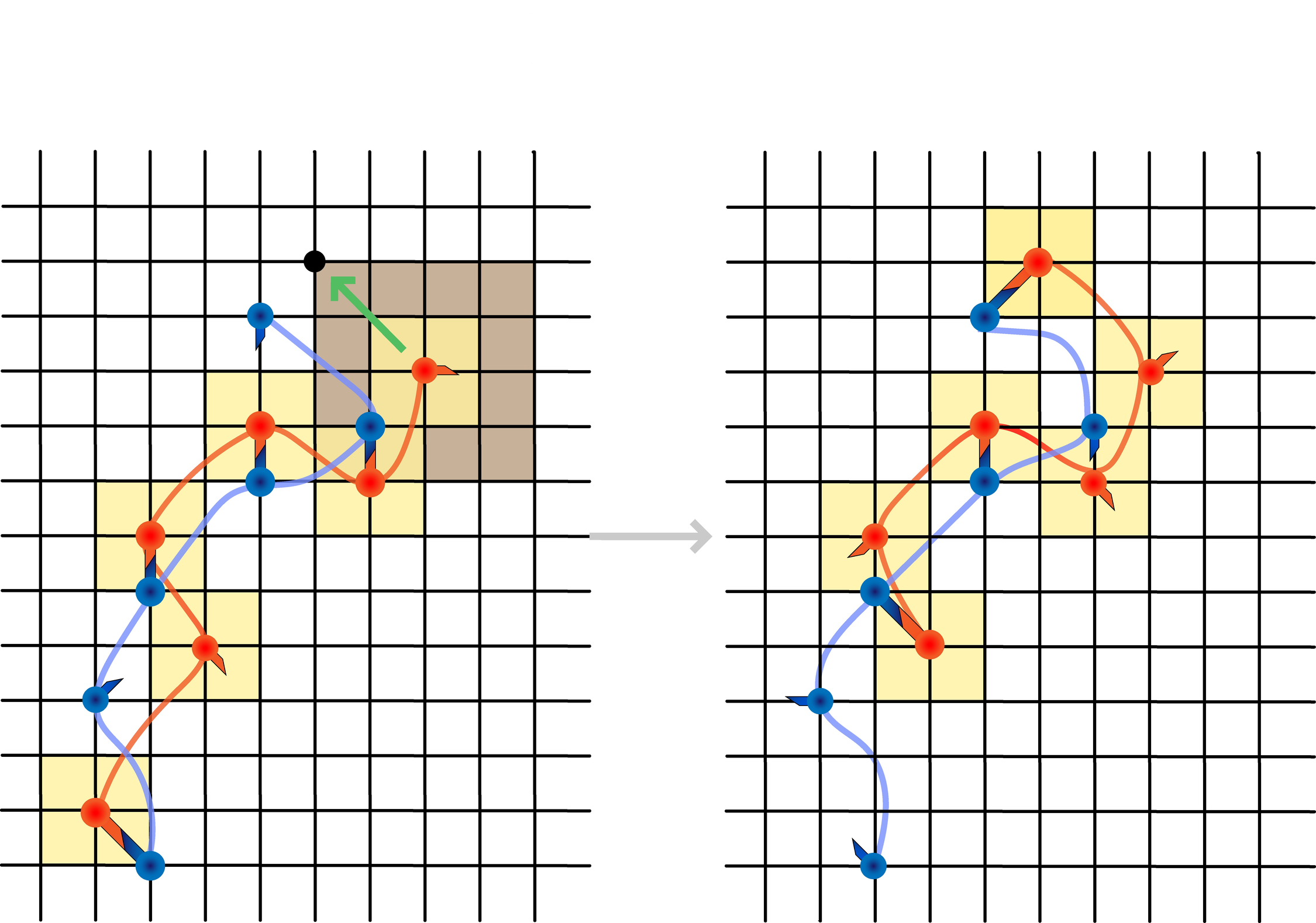

Supplement: S3 Fig — For a given randomly selected chain that has the same linker lengths between each monomer, an end is randomly picked. Then, a version of the local move is performed where the selected end is moved to a new random location that is an empty lattice site within 2 lattice sites in each coordinate (brown box). If an empty site is found within a predetermined number of trials, the number of orientational candidates is calculated for the whole chain in the old and the new configuration (yellow boxes). The modified metropolis criterion is then used to determine if the move is accepted or rejected. Note that since the whole chain is orientationally biased, monomers may have a different orientational state after the move is accepted, as shown in the figure. (TIF) [file pcbi.1007028.s003.tif]

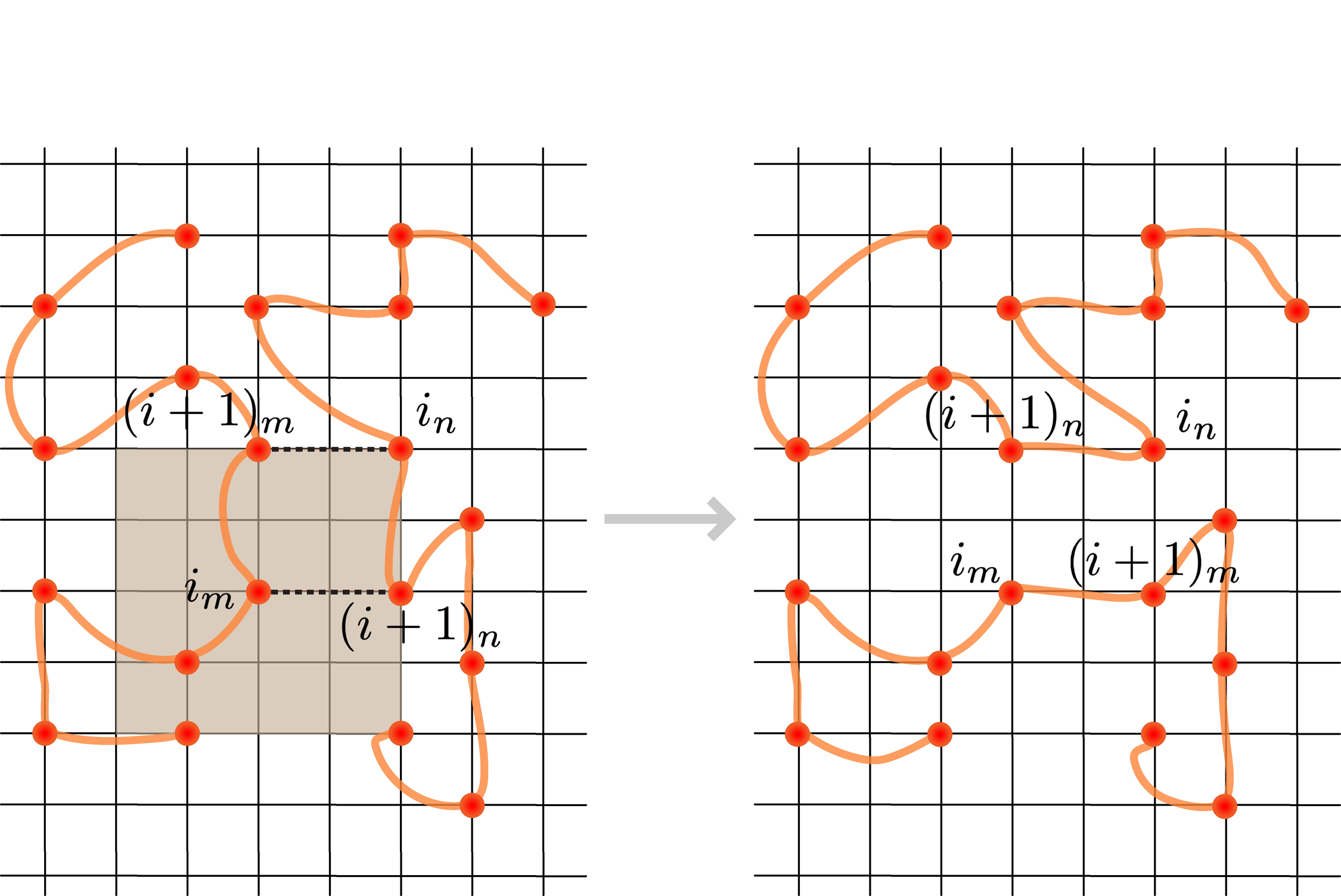

Supplement: S4 Fig — For a randomly selected monomer, a 2×2×2 cube around the monomer is searched for appropriate bridging candidates (brown box), where an appropriate bridging candidate is the next monomer from a different chain, is within a linker length of the selected monomer as shown by the dashed line connecting im and (i+1)n. Furthermore, the distance between (i+1)m and in must also be within a linker length as depicted by the upper dashed line. A list of all possible candidates is calculated and then a randomly chosen candidate is used to break and remake covalent bonds. This results in a large conformational change for both polymers. If the selected polymer is not linear, the move is rejected outright. (TIF) [file pcbi.1007028.s004.tif]

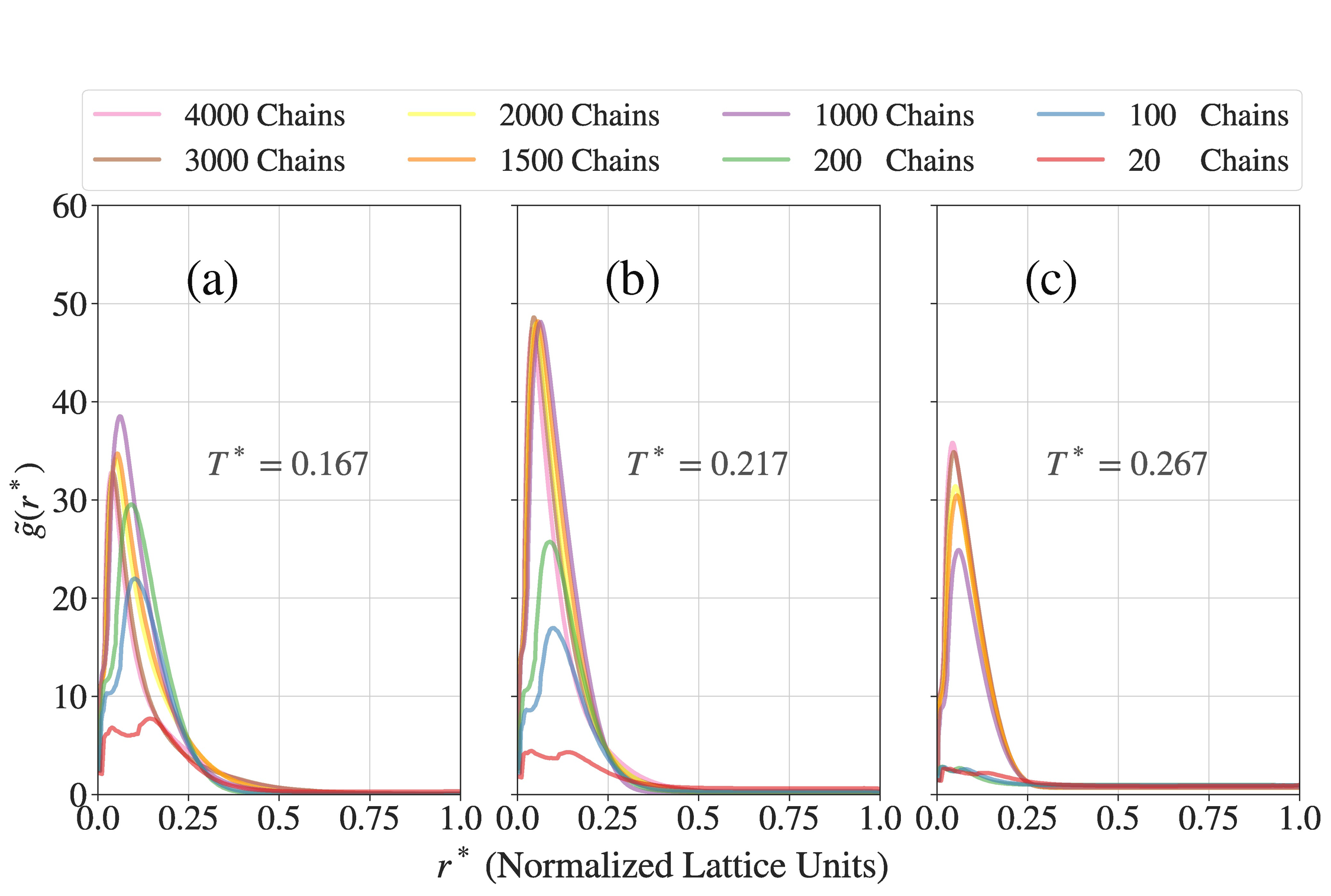

Supplement: S5 Fig — The pair distributions from Figs 9(B) and 11 are used to compute the relevant radial distribution functions. This analysis is relevant because the radial distribution functions are used to extract the value of the order parameter that detects the onset of phase separation. For systems where the number of molecules An + Bn molecules is greater than 200, the radial distribution functions g˜(r*) start to deviate from one another only at the lowest temperatures where broken ergodicity becomes an issue. Therefore, for the An + Bn system studied in this calibration, it appears that the numbers of An + Bn molecules have to be greater than 200 in order to obtain reliable information about the phase behavior. (a) g˜(r*) extracted for different numbers of An and Bn molecules for T* = 0.167. (b) g˜(r*) extracted for different numbers of An and Bn molecules for T* = 0.217. (c) g˜(r*) extracted for different numbers of An and Bn molecules for T* = 0.267. (TIF) [file pcbi.1007028.s005.tif]
